# Aminoxyl Radicals of B/P Frustrated Lewis Pairs: Refinement of the Spin-Hamiltonian Parameters by Field- and Temperature-Dependent Pulsed EPR Spectroscopy

**DOI:** 10.1371/journal.pone.0157944

**Published:** 2016-06-23

**Authors:** Marcos de Oliveira, Robert Knitsch, Muhammad Sajid, Annika Stute, Lisa-Maria Elmer, Gerald Kehr, Gerhard Erker, Claudio J. Magon, Gunnar Jeschke, Hellmut Eckert

**Affiliations:** 1 Instituto de Física de São Carlos, Universidade de São Paulo, P.O. Box 369, 13560-970, São Carlos, São Paulo, Brazil; 2 Institut für Physikalische Chemie, WWU Münster, Corrensstr. 30, D 48149 Münster, Germany; 3 Organisch-Chemisches Institut, WWU Münster, Corrensstr. 40, D 48149 Münster, Germany; 4 Laboratorium für Physikalische Chemie, ETH Zürich, Vladimir-Prelog-Weg 2, 8049 Zürich, Switzerland; Dundee University, UNITED KINGDOM

## Abstract

Q-band and X-band pulsed electron paramagnetic resonance spectroscopic methods (EPR) in the solid state were employed to refine the parameters characterizing the anisotropic interactions present in six nitroxide radicals prepared by N,N addition of NO to various borane-phosphane frustrated Lewis pairs (FLPs). The EPR spectra are characterized by the g-anisotropy as well as by nuclear hyperfine coupling between the unpaired electron and the ^11^B/^10^B, ^14^N and ^31^P nuclear magnetic moments. It was previously shown that continuous-wave spectra measured at X-band frequency (9.5 GHz) are dominated by the magnetic hyperfine coupling to ^14^N and ^31^P, whereas the g-tensor values and the ^11^B hyperfine coupling parameters cannot be refined with high precision from lineshape fitting. On the other hand, the X-band electron spin echo envelope modulation (ESEEM) and hyperfine sublevel correlation (HYSCORE) spectra are completely dominated by the nuclear hyperfine coupling to the ^11^B nuclei, allowing a selective determination of their interaction parameters. In the present work this analysis has been further validated by temperature dependent ESEEM measurements. In addition, pulsed EPR data measured in the Q-band (34 GHz) are reported, which present an entirely different situation: the g-tensor components can be measured with much higher precision, and the ESEEM and HYSCORE spectra contain information about all of the ^10^B, ^11^B, ^14^N and ^31^P hyperfine interaction parameters. Based on these new results, we report here high-accuracy and precision data of the EPR spin Hamiltonian parameters measured on six FLP-NO radical species embedded in their corresponding hydroxylamine host structures. While the ESEEM spectra at Q-band frequency turn out to be very complex (due to the multinuclear contribution to the overall signal) in the HYSCORE experiment the extension over two dimensions renders a better discrimination between the different nuclear species, and the signals arising from hyperfine coupling to ^10^B, ^11^B, ^14^N and ^31^P nuclei can be individually analyzed.

## Introduction

Recent work in the field of organic catalysis has revealed the remarkable reactivity of Frustrated Lewis Pairs (FLP) [1]. FLPs are molecular systems comprising both a Lewis acid and a Lewis base center, which would normally result in the formation of a covalent bond. If both Lewis centers are connected to bulky substituents, however, the covalent interactions between them are reduced (“frustration”), thereby imparting cooperative catalytic activity to the molecule. FLPs are of great interest in catalysis (e.g. for H_2_ activation) and for forming adducts with the ecologically problematic gases (CO, CO_2_, SO_2_, etc.) [2–6]. In particular, intramolecular FLPs containing borane (Lewis acid) and phosphane (Lewis base) centers exhibit remarkable cooperative binding of such small molecules. When exposed to nitric oxide these B/P FLPs form heterocyclic chemically and thermally stable free aminoxyl radicals, which are interesting optical, magnetic and catalytic materials in their own right [5].

In previous work their liquid-state EPR and solid-state NMR properties have been studied in detail [5,7]. We have recently also studied the anisotropic electronic hyperfine interaction tensors in solid samples using DFT calculations and X-band EPR spectroscopy [8]. In principle, the solid-state EPR spectra are expected to be influenced by a multitude of parameters, including the components of the *g*-tensor, the hyperfine coupling tensors describing the interactions with the ^11^B, ^10^B, ^14^N, and ^31^P nuclei, and the electric field gradient tensors (EFG) at the ^11^B, ^10^B and ^14^N nuclear sites. Because of this multitude of interactions, computationally guided simulations are required for the proper analysis of the spectra. In our previous publication we have outlined a feasible strategy for the solid-state EPR characterization of this system at X-band frequency, based on a detailed analysis of continuous-wave EPR lineshapes and ESEEM and HYSCORE spectra, in conjunction with calculations from density functional theory (DFT) [8]. The combination of the DFT calculations with EPR experiments is a powerful tool for the determination of EPR tensor parameters and has been widely explored in the literature [9–15].

For the radicals examined in that work at X-band frequency, we found that the amplitudes of the echo modulations caused by the strongly coupled ^14^N and ^31^P nuclei are considerably lower than those caused by the hyperfine interaction with the ^11^B nuclei. This can be understood to be due to a strong anisotropy of the hyperfine coupling tensor, leading to a wide dispersion of modulation frequencies in powdered samples. As a consequence of the product rule [16], these weaker modulations are suppressed in the experimental spectra [17]. Along similar lines, the ^10^B nucleus experiences a relatively strong quadrupolar interaction (*C*_*Q*_ ∼ 3 MHz), which exceeds the ^10^B nuclear Zeeman frequency at X-band significantly. This renders the ^10^B quadrupolar interaction dominant in the spin Hamiltonian. Again, because of strong anisotropic dispersion effects in polycrystalline samples the ^10^B ESEEM signal becomes undetectable in the presence of the ^11^B based signals [8]. Therefore the previously reported ESEEM and HYSCORE experiments at X-band are completely dominated by the ^11^B spin Hamiltonian, while the cw-EPR spectral lineshapes are mostly affected by the hyperfine interactions with the ^31^P and ^14^N nuclei, making cw and pulsed EPR techniques highly complementary. Unfortunately, the cw spectra of these solid samples do not have enough resolution for an unambiguous determination of the g-tensor components, which influence the EPR lineshape at X-band to a lesser extent. Also, no definitive information regarding the ^14^N quadrupolar coupling could be obtained [8]. In order to validate and/or improve upon the previous results, in the present work we have undertaken a Q-band EPR study for further characterization and refinement of the spin Hamiltonian parameters in these FLPs. At the magnetic field strength (1.25 T) corresponding to the Q-band frequency range, the ^14^N and ^31^P Larmor frequencies are higher, getting closer to the values of the hyperfine coupling constants. In this condition, higher modulation depths for these species are expected so that they may become detectable in the ESEEM and HYSCORE experiments. Furthermore, at the higher field strength the impact of the *g*-anisotropy upon the EPR lineshape will be increased, allowing for a more precise refinement of the *g*-tensor parameters. The results obtained here will be compared with DFT calculations and with our previously published X-band EPR results.

Finally, even though the X-band ESEEM spectra are exclusively affected by the hyperfine coupling with the ^11^B nuclei, their simulation still requires the refinement of nine independent parameters. As such a multi-parameter fitting approach can obviously lead to multiple solutions, the guidance of these simulations by ab-initio calculations proved to be very important [8]. During the course of these studies, we have observed a distinct temperature dependence of the ESEEM spectra. This is not unexpected as molecular and lattice vibrations modulate both the electron-nuclear hyperfine interactions and the local electric field gradients, producing a monotonic decrease of the interaction strength with increasing temperature. This effect can be used for further validation of the ESEEM fitting approach, if the simulation parameters of the experimental data indicate such temperature dependent effects. This question is also examined within the present study.

## Theoretical Aspects of ESEEM Based Experiments

### Three-pulse ESEEM

The standard one-dimensional ESEEM experiment used in the present study consists of a stimulated echo, produced by a three-pulse sequence *(t*_*p*_*)—τ—(t*_*p*_*)—T—(t*_*p*_*)–echo* [16,18]. As *T* is being incremented, nuclear hyperfine and electron-nuclear magnetic dipole-dipole couplings produce a modulation of the echo envelope. The electron coherence order is zero during this time interval, and the modulation thus arises from nuclear coherences [16]. Considering an electron-nucleus *S* = ½, *I* = ½ spin pair with axial hyperfine tensor and isotropic *g*-tensor, and ideal non-selective pulses, the echo envelope modulation formula is given by[16]

$$\begin{array}{l} V_{3p}\left(\tau ,T\right)=1-\frac{k}{4}\left\{\left[1-\text{cos}\left(2\pi \upsilon_{\beta }\tau \right)\right]\left[1-\text{cos}\left(2\pi \upsilon_{\alpha }\left(T+\tau \right)\right)\right]+\left[1-\text{cos}\left(2\pi \upsilon_{\alpha }\tau \right)\right]\left[1-\text{cos}\left(2\pi \upsilon_{\beta }\left(T+\tau \right)\right)\right]\right\},\\ k={\left(\frac{1}{2\pi }\frac{B\nu_{I}}{\upsilon_{\alpha }\upsilon_{\beta }}\right)}^{2}\\ B=\left(A_{∥}-\;A_{⊥}\right)\text{sin}\theta \text{cos}\theta \; \end{array}$$ (1)

The quantities *v*_*α*_ and *v*_*β*_ are the nuclear frequencies that correspond to the two electron spin states *α* (*m*_*S*_ = 1/2) and *β* (*m*_*S*_ = -1/2). *v*_*I*_ is the nuclear Larmor frequency, *A*_⊥_ = *A*_*xx*_ = *A*_*yy*_, *A*_∥_ = *A*_*zz*_, where *A*_*xx*_, *A*_*yy*_, *A*_*zz*_ are the principal values of the nuclear hyperfine coupling tensor, and *θ* is the angle between the magnetic field direction and the *A*-tensor principal axis. The quantity *k*, the so-called modulation depth parameter, defines the amplitude of the modulation. Fourier transformation of the modulation yields the ESEEM spectra. In the limit of low modulation depth these spectra can be approximately described by a superposition of all the nuclear precession frequency components associated with the different nuclei to which the electron spins are coupled.

An important feature of three-pulse ESEEM is the dependence of the modulation amplitude on *τ*, as is clear from the factors 1 –cos(2*πv*_*β*_*τ*) and 1 –cos(2*πv*_*α*_*τ*). For *v*_*α*,*β*_ *= n/τ* (*n = 1*, *2*,*…*), the modulation at the frequency *v*_*β*,*α*_ vanishes. Thus the three-pulse ESEEM spectra are subject to “blind spots”, which become denser for increasing evolution time *τ*. Consequently, the three-pulse ESEEM experiment has to be performed at more than one *τ* value, in order to ensure that such blind spots do not obscure certain frequency components. The chief observable in the ESEEM spectra is the modulation depth parameter *k*, recorded as a function of frequency. For the case of an isotropic hyperfine interaction (*A*_⊥_ = *A*_∥_), or if the magnetic field is oriented along one of the principal axes of the hyperfine tensor (*θ* = 0 or *θ* = π/2), the echo modulation vanishes (*k* = 0), since in these cases the parameter *B* in the above equation becomes zero. The depth parameter *k* also approaches zero in cases of very weak (*v*_*α*_ ≈ *v*_*β*_ ≈ *v*_*I*_ *>> B*) or very strong hyperfine coupling anisotropy (*v*_*I*_ << *v*_*α*_, *v*_*β*_; *B/2* ≤ *v*_*α*_, *v*_*β*_).

### Hyperfine sublevel correlation spectroscopy

The HYSCORE technique is a powerful tool for the investigation of weak hyperfine and nuclear electric quadrupolar couplings of paramagnetic centers in disordered systems. This is a two-dimensional experiment that correlates nuclear transitions in different electron spin manifolds, *α* and *β*. This is achieved by using a π pulse, between the second and the third pulses in the ESEEM sequence, yielding the four-pulse sequence *(t*_*p*_*)—τ—(t*_*p*_*)—t*_*1*_ *- (2t*_*p*_*)—t*_*2*_ *- (t*_*p*_*)–echo* [16,18]. The effect of the π pulse is the transfer of nuclear coherence between the two electron spin manifolds. Therefore, it correlates the frequency *v*_*α*_ of a given nucleus with the frequency *v*_*β*_ of the same nucleus. The frequency in the second dimension is then measured by the introduction of another variable delay *t*_*2*_ between the *π* pulse and the last π/2 pulse that reconverts the nuclear coherence to observable electron coherence. The correlations between nuclear spin transitions manifest themselves in the HYSCORE spectrum as non-diagonal cross-peaks at (*v*_*α*_, *v*_*β*_), (*v*_*β*_, *v*_*α*_) and (-*v*_*α*_, *v*_*β*_), (-*v*_*β*_, *v*_*α*_), respectively in the (+,+) and (-,+) quadrants of the 2D-spectru [16,19]. In the limit of a weak hyperfine interaction (*A* < 2*v*_*I*_), the contributions with positive phase modulation dominate and the cross-peaks appear in the (+,+) quadrant [16,20,21]. In the opposite limit of a strong hyperfine interaction (*A* > 2*v*_*I*_), the contributions with negative phase modulation dominate and the cross-peaks appear mostly in the (-,+) quadrant. Near the cancellation condition (*A*_*iso*_ ~ 2*v*_*I*_), the cross-peaks in the 2D-HYSCORE spectrum have comparable intensities in both quadrants [16,20].

## Materials and Methods

### Sample preparation and characterization

**Fig 1:**
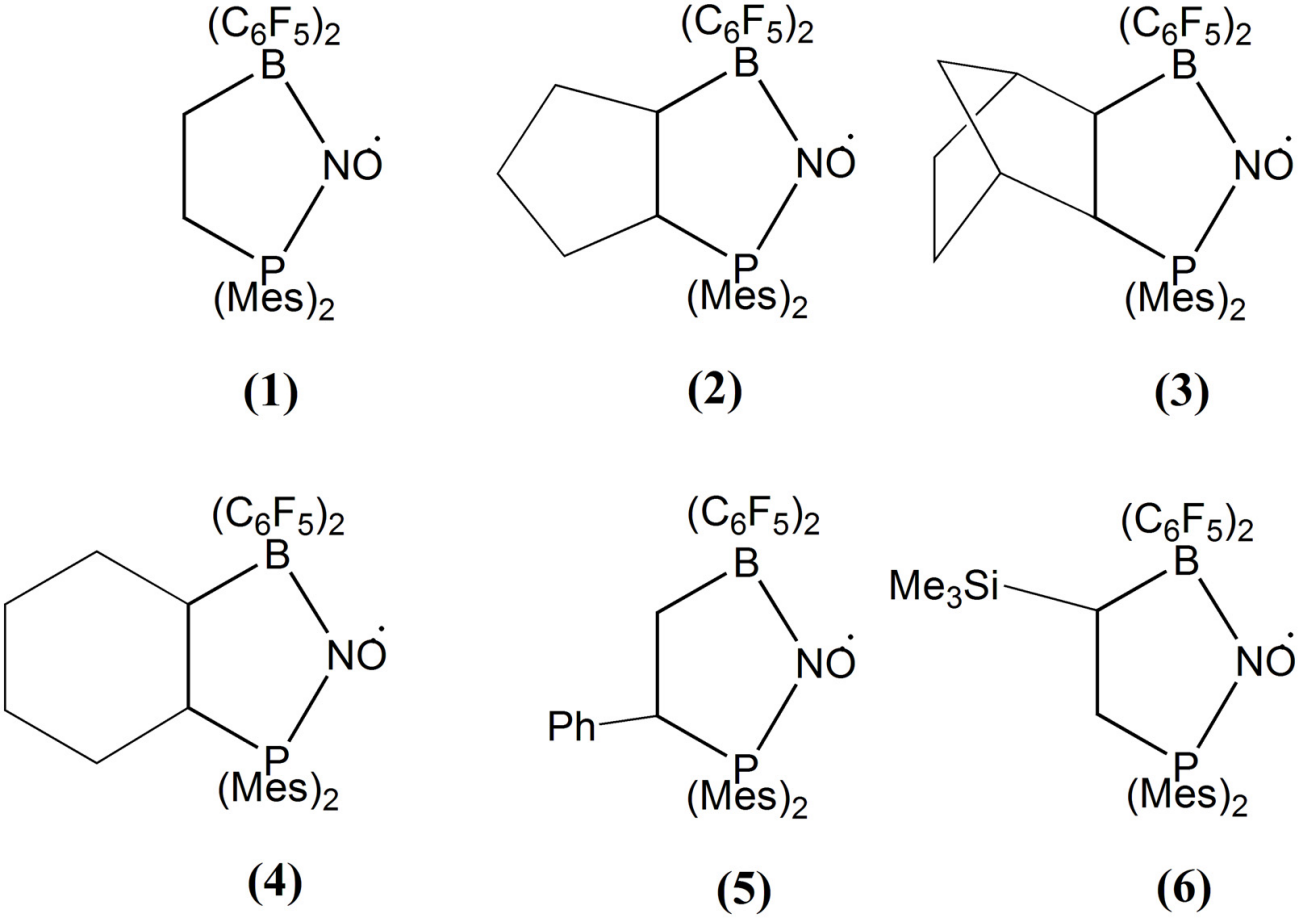
Compounds studied within this work.

The molecular structures of the FLPs under investigation are depicted in Fig 1. They were produced via N,N addition of NO to the parent FLP in fluorobenzene solution at room temperature. Detailed information on their synthesis and characterization is given in the literature [2,5,6,22]. As high radical concentrations lead to a broadening of the EPR spectra due to strong intermolecular exchange interactions, it is necessary to study these molecules in a magnetically dilute state. This was accomplished by a hydrogenation reaction of the radical species with 1,4 cyclohexadiene in benzene solution, yielding the corresponding diamagnetic hydroxylamine compounds. As this reaction is not fully quantitative, the reduction product still contains a small number of the paramagnetic radical molecules. It is reasonable to assume that these radical species are randomly distributed and substitute the hydroxylamine molecules within the crystal structure of the host compound. Previous publications show the crystal structures of the radical species and the FLP-NOH compounds of samples **1** [22], **3** [5], **4, 5** and **6** [6]. the solid-state NMR characterization of sample **3** [5,7], and the solid-state X-band EPR characterization of samples **1**, **2** and **3** [8].

### EPR spectroscopy

The EPR experiments were carried out on E-580 BRUKER Elexsys spectrometers operating at X-band and Q-band frequencies. The Q-band experiments were performed at 100 K. ESEEM spectra were obtained at external field strengths of 1.23 T, using the three-pulse sequence described above, with a π/2 pulse length *t*_*p*_ = 12 ns. The delay between the first and second pulses, *τ*, was set between 100 ns and 380 ns. The time interval *T* was incremented in 8 ns steps starting with *T* = 300 ns; 300 acquisitions were accumulated for each increment with repetition times of 300 *μ*s and up to 20 scans were added up for signal averaging. A four-step phase cycle of the first and second pulse was used for echo detection to avoid unwanted primary echoes and FID distortions [23]. The resulting data were processed in the following way: the modulated echo decay was fitted to a biexponential function, which in turn is subtracted from the experimental data in order to isolate the oscillatory component. Following further apodization and zero-filling, the oscillating signal was Fourier-transformed, resulting in the ESEEM spectrum. The echo detected field-sweep (EDFS) spectra were recorded using the three-pulse sequence. The integrated echo intensities were measured as a function of the magnetic field strength over a range of 1.20–1.25 T. The pulse spacing between the first two pulses (*τ*) was set to 340 ns, and a long evolution time between the second and the third pulse (1 μs) was chosen in order to suppress nuclear modulation effects upon the lineshape.

The HYSCORE experiments were conducted at external magnetic field strengths of 1.23 T, with τ = 340 ns. This τ delay was chosen to avoid blind spots for any of the relevant nuclei in the present sample. The latter was confirmed by additional measurements at τ = 300 ns and 380 ns. The echo intensity was measured as a function of *t*_*1*_ and *t*_*2*_, which were incremented in steps of 8 ns from the initial value of 300 ns. Pulses of *t*_*p*_ = 10 ns length for the π/2 pulse and 2*t*_*p*_ = 20 ns length for the π pulse were used to record a 200×200 data matrix. Following further apodization and zero-filling (to 512×512 points), the oscillating signal was Fourier-transformed in both dimensions, resulting in the HYSCORE spectrum. A 4-step phase cycle was used to eliminate unwanted coherences.

The temperature dependent X-band experiments were performed at a magnetic field strength of 335 mT. All samples were measured over the temperature range from 100 K to 300 K in steps of 50 K using the three-pulse ESEEM sequence described above. At each temperature the ESEEM spectrum was measured at two different *τ*-delays of 170 ns and 250 ns to ensure that no frequency components were suppressed by the blind spots. The time interval *T* was incremented in steps of 16 ns starting at 300 ns. For each increment 100 acquisitions were performed with a repetition time of 200–400 *μ*s, and 20 to 250 scans were added up. The π/2 pulse length was set to 8 ns. Solid-state powder EPR data were simulated by the functions “saffron” and “pepper” of the software package EasySpin [23] implemented in MATLAB (MathWorks, Inc).

## Results and Data Analysis

### Q-band echo detected field sweep

**Fig 2:**
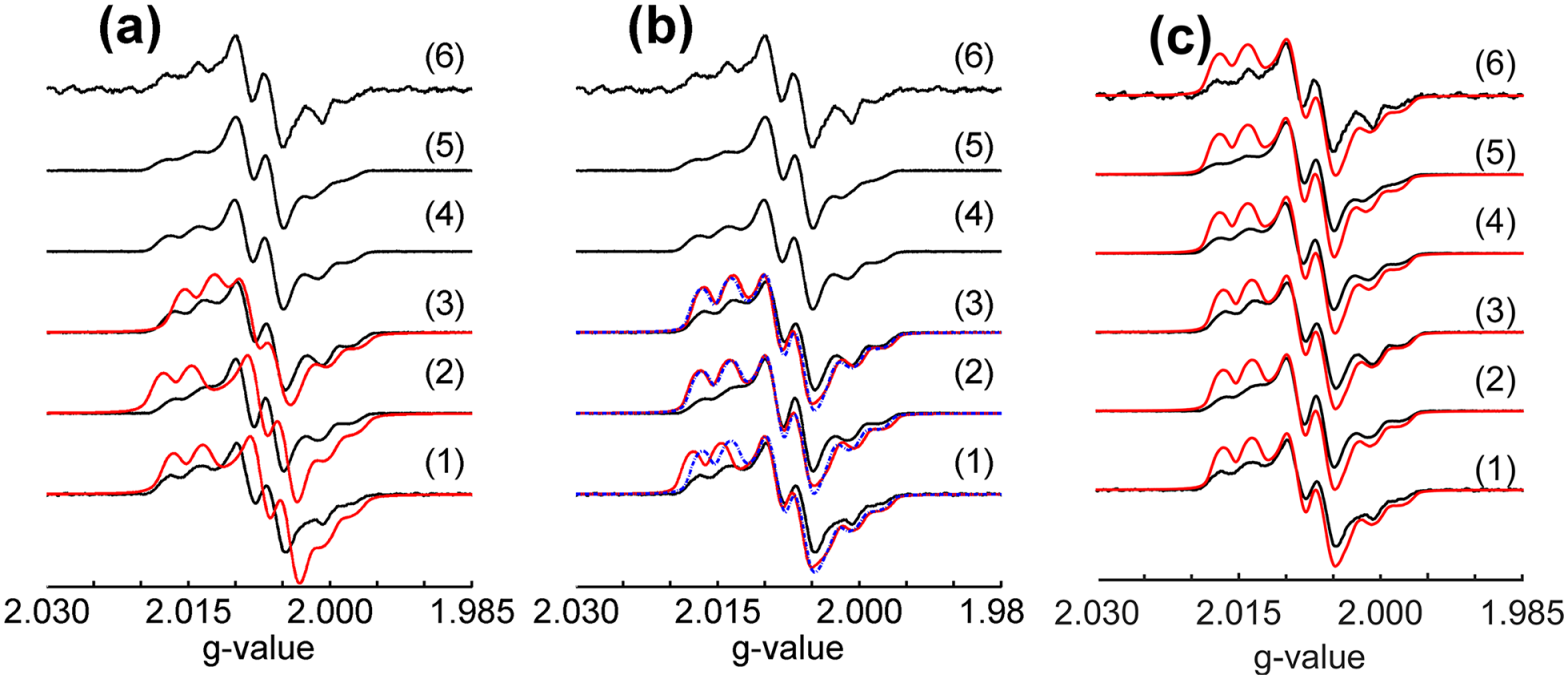
First derivative Q-band EPR spectra computed from experimental echo detected field-sweep spectra (black curves) for the set of FLP adducts. Red and dotted blue curves are EasySpin [23] simulations considering different sets of parameters as follows: (a) simulations (red curves) considering “best fit” parameters from the previous X-band results [8]; (b) red curves—simulations based on the DFT-calculated parameters from Ref. [8]; blue curves—simulations based on the DFT-calculated parameters and subsequent adjustments of the *g*-tensor principal values, as shown in Table 1; and (c) simulations considering the best-fit parameters shown on the third column of Table 1, followed by adjustment of the anisotropic *g*-values (see Table 1). For samples **1** to **3** the Euler angles for the different tensors were taken from DFT calculations (Ref. [8]). For samples **4** to **6** DFT calculations were not performed and the set of Euler angles calculated for sample **1** was used in the simulations.

Owing to the high spin dilution of the samples studied in this work and saturation of the signal at low temperatures already for low microwave powers, cw-EPR experiments at Q-band were hampered by low signal-to-noise ratios. Therefore, all the EPR spectral analyses are done on EDFS spectra. Fig 2 shows the corresponding first derivatives for samples **1–6** (black curves) and EasySpin [23] simulations obtained for selected samples and parameter sets (red and dashed blue curves). We consider three separate sets of simulations. Fig 2(a) shows the simulations based on our previous X-band study [8]. Fig 2(b) shows the simulations obtained from previous DFT calculations [8], followed by optimization of the anisotropic *g* tensor parameters. Finally, Fig 2(c) shows the simulations obtained from the Q-band HYSCORE experiment of the present study (see further discussion below), followed by the optimization of the anisotropic g-parameters. Table 1 lists the parameters resulting from these three simulation approaches. In this table, we use the notation

$$\begin{array}{l} A_{iso}=\frac{1}{3}\left(A_{xx}+A_{yy}+A_{zz}\right)\\ \delta_{A}=A_{33}-A_{iso}\\ \eta_{A}=\;\frac{\left(A_{11}-A_{22}\right)}{\delta } \end{array}$$ (2)

to describe the isotropic value, the anisotropy and the asymmetry parameter of the hyperfine coupling tensors with the nuclei ^14^N, ^31^P, ^11^B and ^10^B. In eq (2) the parameters *δ*_*A*_ and *η*_A_ are defined from the hyperfine tensor components *A*_11_, *A*_22_ and *A*_33_ using the Haeberlen convention |*A*_33_—*A*_*iso*_| > |*A*_11_—*A*_*iso*_| > |*A*_22_—*A*_*iso*_|. Table 1 also lists the nuclear electric quadrupole tensor parameters *C*_*Q*_ (product of nuclear electric quadrupole moment and the principal value of the electric field gradient tensor *eq*_*zz*_) and the electric field gradient asymmetry parameter *η*_*Q*_ = (*eq*_xx_-*eq*_yy_)/*eq*_zz_ defining the deviation of the electric field gradient from cylindrical symmetry.

**Table 1:** Hyperfine (*A*-), Quadrupole (*Q*-) and *g*-tensor principal values for the set of compounds studied in this work obtained by DFT calculations [8] and from X-band [8] and Q-band EPR analyses. The conventions *A*_*xx*_ < *A*_*yy*_ < *A*_*zz*_ and *g*_*xx*_ > *g*_*yy*_ > *g*_*zz*_ are followed. The parameters *δ*_*A*_ an *η*_*A*_ are calculated according to Eq (2). The DFT calculations were done on geometry-optimized structures from the gas phase on a TPSS-D3/def2-TZVP level, the *A*-tensors were calculated on a B3LYP/TZ2P level [8].

Parameters / Method		X-band			DFT			Q-band (best fit)					
		1	2	3	1	2	3	1	2	3	4	5	6
***A*(**^**14**^**N) (MHz)**	***A***_***x***_ **(± 0.1)**	-4.1	-1.1	-5.3	-4.0	-3.4	-3.0	-0.1	-0.1	-0.1	-0.1	-0.1	-0.1
	***A***_***y***_ **(± 0.1)**	-3.6	-0.5	-4.7	-3.3	-2.8	-2.5	0.5	0.5	0.5	0.5	0.5	0.5
	***A***_***z***_ **(± 1)**	52.2	58.7	61	53.5	56.4	58	56.6	56.6	56.6	56.6	56.6	56.6
	***A***_***is***_ **(± 1)**	15	19	17	15	17	18	19	19	19	19	19	19
	***δ***_**A**_ **(± 0.2)**	37.4	39.7	44.0	38.1	39.7	40.6	37.6	37.6	37.6	37.6	37.6	37.6
	***η***_**A**_ **(± 0.01)**	0.01	0.02	0.01	0.02	0.02	0.01	0.02	0.02	0.02	0.02	0.02	0.02
***A*(**^**31**^**P) (MHz)**	***A***_***xx***_ **(± 1)**	-57	-57	-61	-55	-57	-57	-55	-55	-55	-55	-55	-55
	***A***_***yy***_ **(± 1)**	-51	-51	-45	-48	-49	-48	-51	-51	-51	-51	-51	-51
	***A***_***zz***_ **(± 1)**	-45	-43	-41	-42	-43	-43	-44	-44	-44	-44	-44	-44
	***A***_***iso***_ **(± 1)**	-51	-50	-49	-48	-50	-49	-50	-50	-50	-50	-50	-50
	***δ***_**A**_ **(± 1)**	6	7	-12	-7	-7	-8	6	6	6	6	6	6
	***η***_**A**_ **(± 0.1)**	1.0	0.8	0.3	0.9	0.8	0.7	0.7	0.7	0.7	0.7	0.7	0.7
***A*(**^**11**^**B) (MHz)**	***A***_***xx***_ **(± 1)**	-11	-11	-11	-11	-11	-11	-11	-11	-11	-11	-11	-11
	***A***_***yy***_ **(± 1)**	-11	-11	-11	-11	-11	-11	-11	-11	-11	-11	-11	-11
	***A***_***zz***_ **(± 0.1)**	-6.7	-6.5	-6.3	-6.6	-6.5	-6.2	-6.3	-6.3	-6.3	-6.3	-6.3	-6.3
	***A***_**iso**_ **(± 0.1)**	-9.6	-9.5	-9.4	-9.5	-9.5	-9.4	-9.4	-9.4	-9.4	-9.4	-9.4	-9.4
	***δ***_**A**_ **(± 0.3)**	2.9	3	3.1	2.9	3	3.2	3.1	3.1	3.1	3.1	3.1	3.1
	***η***_**A**_ **(± 0.1)**	0.0	0.0	0.0	0.0	0.0	0.0	0.0	0.0	0.0	0.0	0.0	0.0
**Q(**^**14**^**N) (MHz)**	***C***_**Q**_ **(± 0.5)**	-	-	-	3.1	2.9	3.3	3.5	3.5	3.5	3.5	3.5	3.5
	***η***_**Q**_ **(± 0.05)**	-	-	-	0.68	0.75	0.88	0.68	0.68	0.68	0.68	0.68	0.68
**Q(**^**11**^**B) (MHz)**	***C***_**Q**_ **(± 0.1)**	-	1.3	1.0	1.3	1.4	1.3	1.0	1.0	1.0	1.0	1.0	1.0
	***η***_**Q**_ **(± 0.02)**	-	0.47	0.57	0.57	0.53	0.65	0.57	0.57	0.57	0.57	0.57	0.57
***g***_***xx***_		2.0150	2.0161	2.0138	2.0161	2.0153	2.0149	2.0151	2.0151	2.0151	2.0155	2.0155	2.0155
***g***_***yy***_		2.0052	2.0055	2.0065	2.0072	2.0071	2.0072	2.0070	2.0070	2.0070	2.0070	2.0070	2.0070
***g***_***zz***_		2.0019	2.0018	2.0018	2.0019	2.0055	2.0019	2.0022	2.0024	2.0022	2.0025	2.0028	2.0024

Fig 2a compares the experimental EDFS spectra (presented in the derivative mode) with simulations obtained previously for the X-band cw spectra of compounds **1–3** [8]. It is evident that these simulations do not reproduce the Q-band EDFS spectra very well, indicating that the set of *g*-parameters deduced from the X-band spectra in reference 8 is not sufficiently accurate. This is understandable, as the main features of those spectra are dominated by the anisotropic magnetic hyperfine interactions with the ^14^N and ^31^P nuclei. As the impact of the anisotropic *g*-tensor upon the EPR lineshape scales linearly with the magnetic field strength, the Q-band spectra are much more sensitive to *g*-tensor variations than the X-band spectra, allowing a refinement of these parameters.

A good starting point for this refinement are the EPR parameters obtained for samples **1–3** from the single molecule DFT calculations reported in Ref. [8] (red curves in Fig 2(b)). Although the relative intensities of the various line shape features are not well matched in these simulations, the peak positions are already reproduced very well. Better agreement between experimental and simulated lineshape features can be obtained by adjusting the *g*-tensor principal values (see Table 1). The resulting simulations are the dotted blue curves in Fig 2(b). Fig 2(c) summarizes a third simulation approach used in the present study, based on more precise values of the ^14^N and ^31^P hyperfine interaction tensor parameters and the ^14^N nuclear electric quadrupolar coupling tensor available from the Q-band HYSCORE data reported in the present study (see detailed discussion below), followed by further optimization of the *g*-tensor values. Since no DFT calculations were done for samples **4–6**, the relative orientations between the interaction tensors were taken to be identical to those determined for sample **1**. This approach is justified by the fact that the previous calculations in reference 8 revealed that the mutual tensor orientations in compounds **1–3** are rather similar. Furthermore, only minor variations in the spectral line shapes are observed when varying the Euler angles relating the tensors. As illustrated in Fig 2c, the simulation approach described above results in the best agreement between experimental and simulated results. In judging the quality of the agreement between the experimental and simulated data we have to bear in mind that the experimental spectra shown in Fig 2 are the first derivatives of echo-detected field sweep spectra, which means that the relative intensities of the various spectral features are influenced by transverse and longitudinal relaxation effects. As these effects are not accounted for in the EasySpin simulations, the goodness of the fit was primarily judged from the match in the frequency positions of the various spectral features rather than their intensities.

### Q-band Hyperfine Sublevel Correlation Spectroscopy

**Fig 3:**
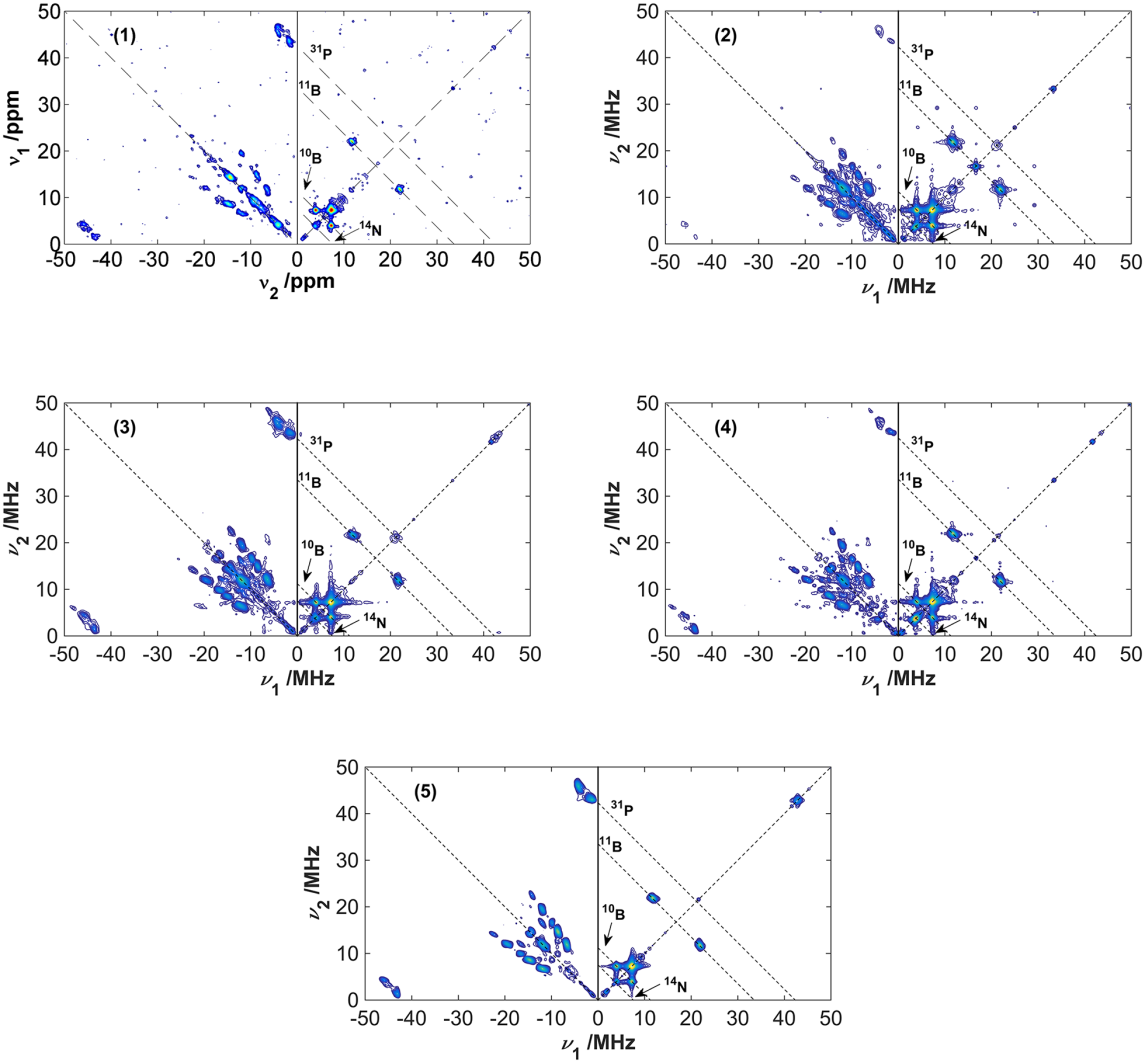
2D-HYSCORE spectra recorded at a magnetic field strength of 1.23 T for the set of FLP samples 1–5. The anti-diagonal dashed lines cross the diagonal at the Larmor frequencies for the isotopes related in the plots. The diagonal peaks at around 34 MHz and 42 MHz are artifacts from the spectrometer.

Fig 3 shows the Q-band HYSCORE spectra obtained for samples **1–5**. No HYSCORE results could be measured for sample **6** as the amount of sample was too small for obtaining a satisfactory signal-to-noise ratio within a reasonable amount of measurement time. For all the samples the HYSCORE spectra result from modulations due to the hyperfine couplings with the ^11^B, ^10^B, ^31^P and ^14^N nuclei. The anti-diagonal lines in Fig 3 cross the diagonal line defined by the nuclear Larmor frequencies. Resonances in the weak coupling limit (|*A*_*iso*_| < 2*v*_I_) for ^11^B, ^10^B and ^14^N are present in the (+ +) quadrant of all the HYSCORE spectra. For samples **2** and **3** a peak at the Larmor frequency of ^31^P is also visible, which may be attributed to a weak dipolar interaction of the electron with the nucleus of a neighboring molecule. In the (- +) section of the spectra, resonance signals in the strong coupling limit (|*A*_*iso*_| > 2*v*_*I*_) are observed for ^14^N (set of peaks closer to the diagonal in the frequency range 10–20 MHz) and ^31^P (signals around *v*_*1*_,*-v*_*2*_ and *v*_*2*_,*-v*_*1*_ with *v*_*1*_ = 45 MHz and *v*_*2*_ = 4 MHz).

Simulations considering electron spins interacting solely with the ^31^P or ^14^N isotopes (S1 to S5 Figs) were performed in order to identify the signals corresponding to each of the nuclei, to explore the sensitivity of various spectral features toward variations of the parameters *A*_*iso*_ (^31^P), *δ*_*A*_(^31^P), *η*_*A*_(^31^P), *A*_*iso*_(^14^N), *δ*_*A*_(^14^N), *η*_*A*_(^14^N) and *C*_*Q*_(^14^N) parameters. As the “best-fit” criterion we used the optimum agreement between experimental and simulated cross-peak positions, as discussed in S1 Text and S6 and S7 Figs. It was further confirmed that no additional HYSCORE peaks were produced by three-spin effects (arising from the simultaneous interaction of the unpaired electron with ^31^P and ^14^N). These simulations clearly show that the spectral regions signifying the effect of ^14^N and ^31^P are very distinct, allowing the unambiguous determination of the EPR parameters by comparison between the simulated and experimental spectra. This was done by performing systematic simulations in which only one EPR parameter was varied, while keeping all the other parameters unchanged. S1 and S2 Figs show these simulations as a function of the ^31^P hyperfine tensor parameters *A*_*iso*_ and *δ*_*A*_. The parameter values that best agree with the experimental data are *A*_*iso*_ = -50 ± 1 MHz and δ_A_ = 6 ± 1 MHz. The same was done for the ^14^N hyperfine parameters in S3 and S4 Figs, resulting in best-fit parameters of *A*_*iso*_ = 19 ± 1 MHz and *δ*_*A*_ = 37.6 ± 0.2 MHz. S5 Fig shows simulations varying the ^14^N quadrupolar coupling constant *C*_*Q*_. In all cases a strong dependence of the HYSCORE data on the EPR and nuclear interaction parameters is observed, which validates the results obtained from the present analysis and allows error estimations as to the precision of the simulation parameters.

**Fig 4:**
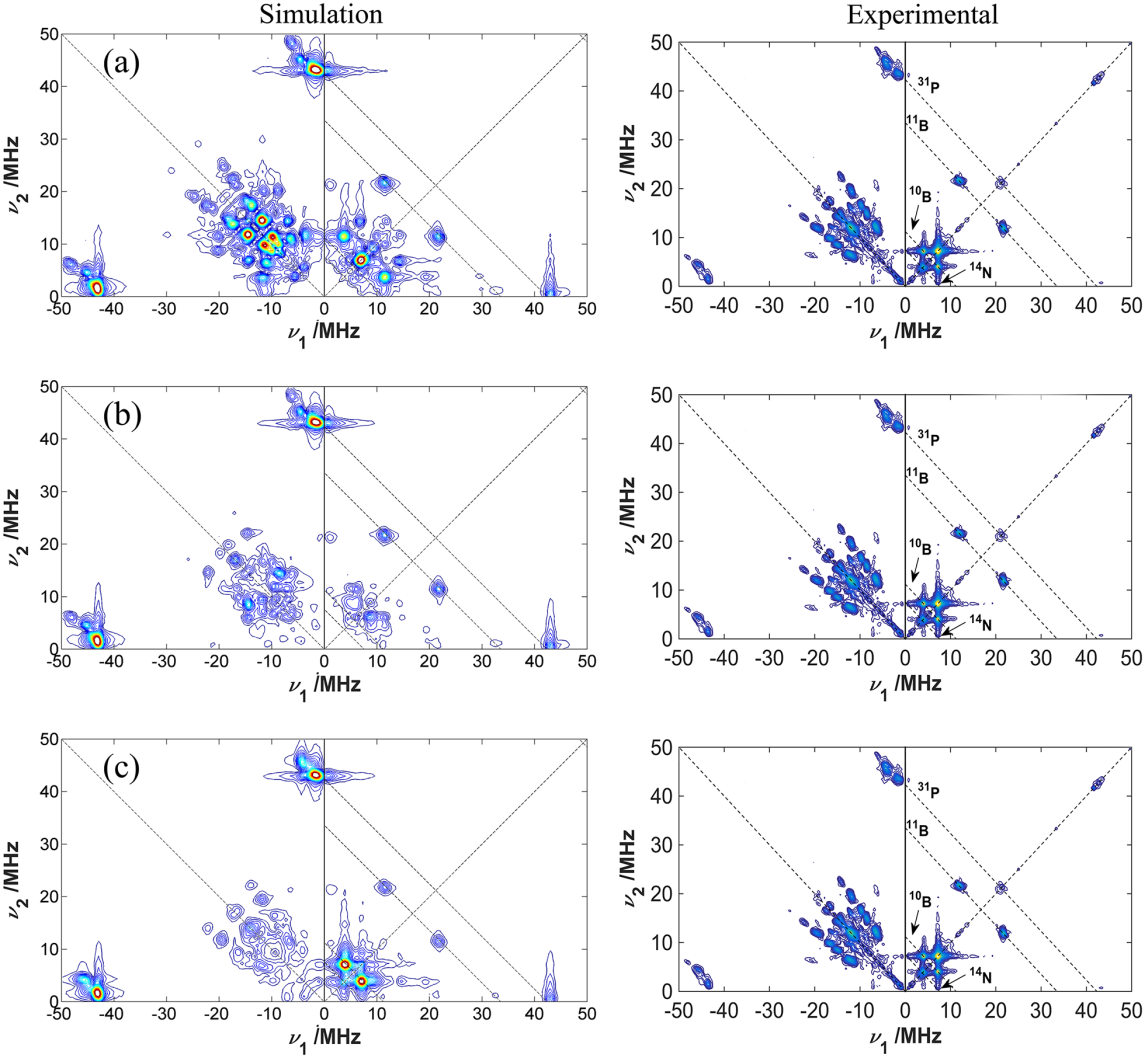
2D-HYSCORE simulated spectra (left) compared with HYSCORE experimental spectrum for sample 3 (right). (a) parameters obtained from DFT calculations [8], (b) parameters extracted from X-band EPR analysis [8], and (c) optimized parameters. The simulations were performed at Q-band frequencies and a magnetic field of 1.23 T. Table 1 shows the set of EPR parameters used in the simulations. The diagonal peaks at around 34 MHz and 42 MHz are artifacts from the spectrometer.

Fig 4 compares simulated and experimental HYSCORE spectra (given for compound **3** as an example), using again the three simulation approaches described for the EDFS spectra of Fig 2. Fig 4a shows a simulated HYSCORE spectrum based on optimized parameters from X-band simulations [8]. Although this set of parameters had reproduced very well the X-band cw and ESEEM spectra, this is clearly not the case for the Q-band experiments. Such deviations were to be expected, since the ^14^N and ^31^P hyperfine coupling parameters had to be extracted from a multiple parameter-fit to the X-band cw spectra before. Fig 4b shows a simulated HYSCORE spectrum based on the DFT-calculated parameters for sample **3** [8]. This result shows that, while the EDFS simulations based on the DFT-calculated parameters alone (Fig 2b) are already in good agreement with the experimental data, the HYSCORE simulations show that optimizations in the ^14^N and ^31^P hyperfine interaction parameters and in the ^14^N quadrupolar coupling parameters still would result in considerable improvement. The final result is shown in Fig 4c, revealing excellent agreement. Due to the lack of peak definition for the ^11^B and ^10^B signals, the parameters used in the simulations in Fig 4a and 4c were taken directly from the ones previously reported in Ref. [8], obtained from the X-band ESEEM and HYSCORE techniques (the ^10^B hyperfine coupling and quadrupolar coupling parameters were scaled from the ^11^B values appropriately). The parameters used for the optimized HYSCORE simulations are summarized in the third column of Table 1. We attempted to develop some numerical criteria for judging the goodness of the fit. For the hyperfine parameters *A*_iso_(^31^P) and *δ*_A_(^31^P) we were able to do so by comparing the root mean square deviations (rmsd) between the experimental and simulated spectra within those spectral regions in the HYSCORE spectra that are dominated by the hyperfine interaction with the ^31^P nuclei. The same rmsd minima were observed for all the compounds measured. For the interaction parameters of the ^14^N nuclei this approach was not successful, however, as the spectra were found to be much more complex. In this case, we had to resort to visual comparison of the simulations with the experimental data in the relevant areas of the contour plots for arriving at the best-fit parameters. In doing so, we focused on the correct reproduction of the peak positions rather than their intensities, as the latter are additionally influenced by relaxation processes that are not accounted for in the simulations. The uncertainties in Tables 1 and 2 were estimated by taking into account the sensitivity of the simulated lineshapes due to changes in the simulation parameters. We obtain ^14^N quadrupolar coupling parameters given by *C*_*Q*_ = 3.5 ± 0.5 MHz and *η*_*Q*_ = 0.68 ± 0.10, close to the values calculated from DFT, *C*_*Q*_ = 2.9 MHz and *η*_*Q*_ = 0.75. The HYSCORE spectra for all the samples are very similar, the differences among them being smaller than the experimental error incurred with the comparison between simulated and experimental data. Therefore, the set of parameters obtained from HYSCORE for sample **3** can be considered as representative for all the samples of the present study. As shown below, an excellent fit to the X-band cw-EPR spectrum of **1–3** can be obtained based on the improved EPR parameter values extracted from the simulation of the Q-band HYSCORE spectra. Fits of similar quality could be obtained for the other compounds as well.

**Table 2:** EPR interaction parameters used for the X-band ESEEM simulations for samples 2–5. *A*_*iso*_, *δ*_*A*_ and *η*_*A*_ are respectively the isotropic and anisotropy and asymmetry parameters of the ^11^B hyperfine coupling tensor, according to the notation given in Eq 2. *α*, *β* and *γ* are the Euler angles, according to notation from Ref. [16], and *C*_*Q*_ and *η*_*Q*_ are the ^11^B nuclear electric quadrupole coupling constant and the EFG asymmetry parameter.

Sample	*T* (K)	*A*_*iso*_ (± 0.02 MHz)	*δ*_*A*_ (± 0.01 MHz)	*η*_*A*_ ± 0.02	*α* ± 10	*β* ± 5	*γ* ± 10	*C*_*Q*_ (± 0.03 MHz)	*η*_*Q*_ ± 0.05
**2**	300	-9.69	3.22	0.05	-40	173	15	1.00	0.50
	250	-9.72	3.22	0.05	-40	173	15	1.03	0.50
	200	-9.78	3.24	0.05	-50	173	15	1.05	0.50
	150	-9.83	3.26	0.05	-60	171	15	1.10	0.45
	100	-9.84	3.26	0.05	-70	169	15	1.12	0.50
**3**	300	-9.27	3.16	0.08	-30	175	20	1.20	0.50
	250	-9.31	3.16	0.08	-40	174	20	1.20	0.50
	200	-9.36	3.16	0.07	-40	173	20	1.22	0.50
	150	-9.40	3.16	0.07	-50	173	20	1.25	0.50
	100	-9.42	3.16	0.07	-50	173	10	1.28	0.50
**4**	300	-9.64	3.24	0.05	-20	173	15	1.10	0.50
	250	-9.66	3.24	0.05	-30	173	15	1.11	0.50
	200	-9.69	3.24	0.05	-30	173	15	1.13	0.50
	150	-9.72	3.24	0.05	-30	173	15	1.14	0.50
	100	-9.74	3.24	0.05	-30	173	15	1.14	0.50
**5**	300	-9.73	3.22	0.04	-20	173	15	1.09	0.50
	250	-9.79	3.24	0.04	-30	172	15	1.10	0.50
	200	-9.83	3.24	0.04	-50	170	15	1.12	0.50
	150	-9.86	3.24	0.04	-60	168	15	1.16	0.45
	100	-9.89	3.20	0.04	-70	166	15	1.21	0.50

### Q-band Electron Spin Echo Envelope Modulation

**Fig 5:**
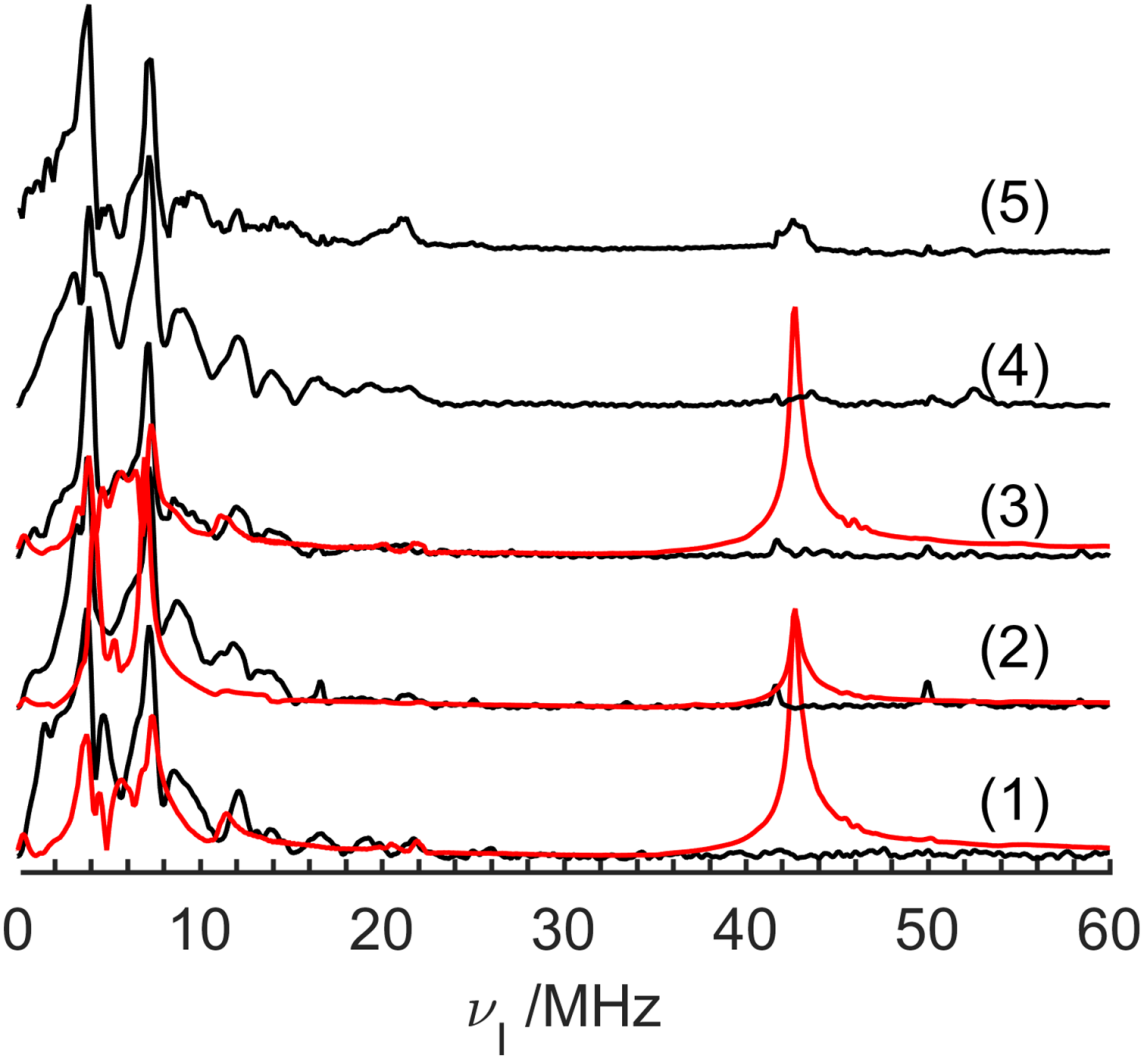
Experimental Q-band ESEEM spectra for the samples 1–5 (black curves). Red curves are EasySpin simulations considering “best fit” principal values of the interaction tensors obtained from the HYSCORE results (see Table 1) and Euler angles from DFT calculations [8]. The asterisk marks indicate frequency positions where spectrometer artifacts are present (narrow peaks).

**Fig 6:**
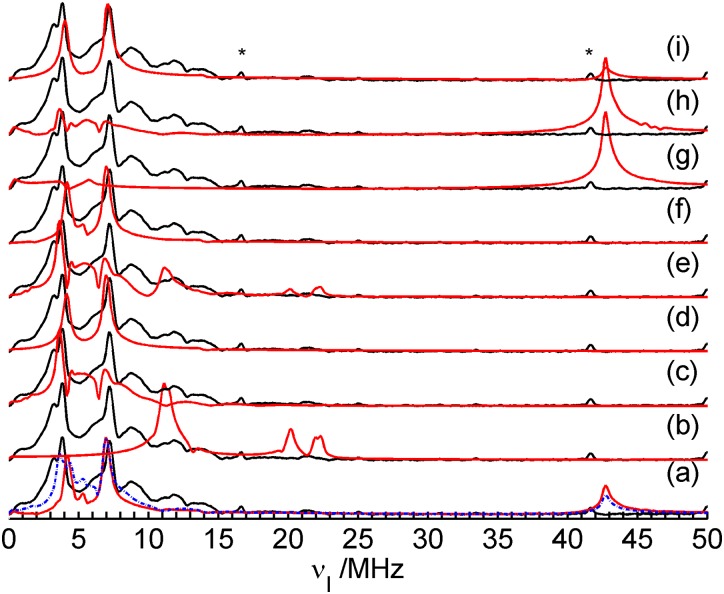
Simulated (red curves) and experimental (black curves) Q-band ESEEM spectra for sample 2. The simulations consider different sets of nuclear species interacting with a single unpaired electron. (a) ^10^B, ^11^B, ^14^N and ^31^P nuclei (red curve); (b) ^11^B nucleus only; (c) ^14^N nucleus only; (d) ^10^B nucleus only; (e) ^11^B and ^14^N nuclei; (f) ^10^B and ^14^N nuclei; (g) ^31^P nucleus only; (h) ^31^P and ^14^N nuclei; (i) ^31^P and ^10^B nuclei. Spectra are internally normalized by the maximum intensity. The asterisk marks indicate frequency positions where spectrometer artifacts are present (narrow peaks). The blue dashed curve in (a) shows an optimized simulation including the interactions with all the nuclei and emphasizing the ^14^N contribution (see text).

Fig 5 shows the ESEEM spectra obtained for the samples **1**–**5** (black curves). No ESEEM signal could be measured for sample **6**. Compared to the ESEEM spectra obtained at the X-band, which are entirely dominated by the hyperfine coupling to ^11^B (in addition to weak dipolar couplings to ^1^H and ^19^F), the Q-band ESEEM spectra are substantially more complicated, revealing distinct contributions from interactions with ^14^N, ^10^B and ^11^B. Also, no weak couplings with ^1^H and ^19^F are detectable here. The experimental data for samples **1–3** are compared with simulations (red curves), based on the parameters previously obtained from the HYSCORE analysis. Two four-spin-system simulations were done in order to obtain each simulated spectrum shown in Fig 5. The first one considers one electron interacting with ^11^B, ^14^N and ^31^P isotopes, while in the second one ^11^B was replaced by ^10^B. Both simulations were then co-added with the weighting factors of the ^11^B and ^10^B natural abundances. For signal attribution, different simulations considering the interaction of the unpaired electrons with different sets of nuclear species were carried out. Fig 6 shows these simulations (red curves) compared with the experimental spectrum of sample **2** (this experimental spectrum was chosen because the highest signal-to-noise ratio realized). Fig 6a reproduces the simulation of Fig 5b for the sake of comparison. Fig 6b shows a simulation that takes into account only interactions with the ^11^B nucleus. This simulation suggests that the feature around 11.5 MHz observed in the experimental data arises from the hyperfine interaction with the ^11^B nuclei. Fig 6c shows a simulation considering only the interaction with the ^14^N nuclei. The ^14^N signal extends over a wide range, with a stronger contribution around 3.7 MHz, coinciding with the experimental peak observed in the same position. Fig 6d shows a simulation considering only interaction with ^10^B nuclei, which shows that the main contribution from this nucleus is the signal near 7 MHz. In order to seek for possible combination peaks that can appear in systems with more than one nuclear spin [17], two simulations considering two nuclear species, ^11^B/^14^N and ^10^B/^14^N were also performed (Fig 6e and 6f, respectively). These latter simulations resulted in spectra that are the simple sum of the spectra for the two isolated systems, i.e. no strong combination peaks are present. Simulations considering ^31^P, isolated (Fig 6g) or combined with ^14^N (Fig 6h) or ^10^B nuclei (Fig 6i) show a peak at around 43 MHz corresponding to the ^31^P modulations. This signal has no correspondence in the experimental data, suggesting a short transversal relaxation time for the ^31^P species. In summary, the Q-band ESEEM spectra seem to be dominated by the responses of the ^14^N, ^11^B and ^10^B nuclei. Unfortunately, some of the minor features observed in the overall spectra cannot be well reproduced by these simulations, and the origin of these signals is still under investigation. The same problem may also occur with the HYSCORE data, but is visually less evident in the contour plots. As the latter emphasize the spectral features with the strongest intensities, it is generally easier to find a good match between experimental and simulated data.

Besides this lack of attribution mentioned above, we suspect that the amplitudes of the different nuclear contributions to the ESEEM spectra are also affected by differences in relaxation times between the nuclear species, which is not taken into account in the simulations. Such lineshape distortions can occur because of the intrinsic dead-time present in the ESEEM experiments, which leads to differences in dephasing rates between signals from different nuclear species. One way of circumventing this problem is to add spectra from different simulations with different weighting factors in order to reproduce the mismatch of the relative intensities of signals from different nuclear species. In the present case, the combination that best approximates the experimental data is the sum of the simulations in Fig 6a (all nuclei) and Fig 6c (^14^N). The result is shown as the blue dashed curve in Fig 6a. Although the resulting spectrum is still not in perfect agreement with the experimental data, it suggests that the ^14^N transversal relaxation time may be longer than those of the other nuclear species.

**Fig 7:**
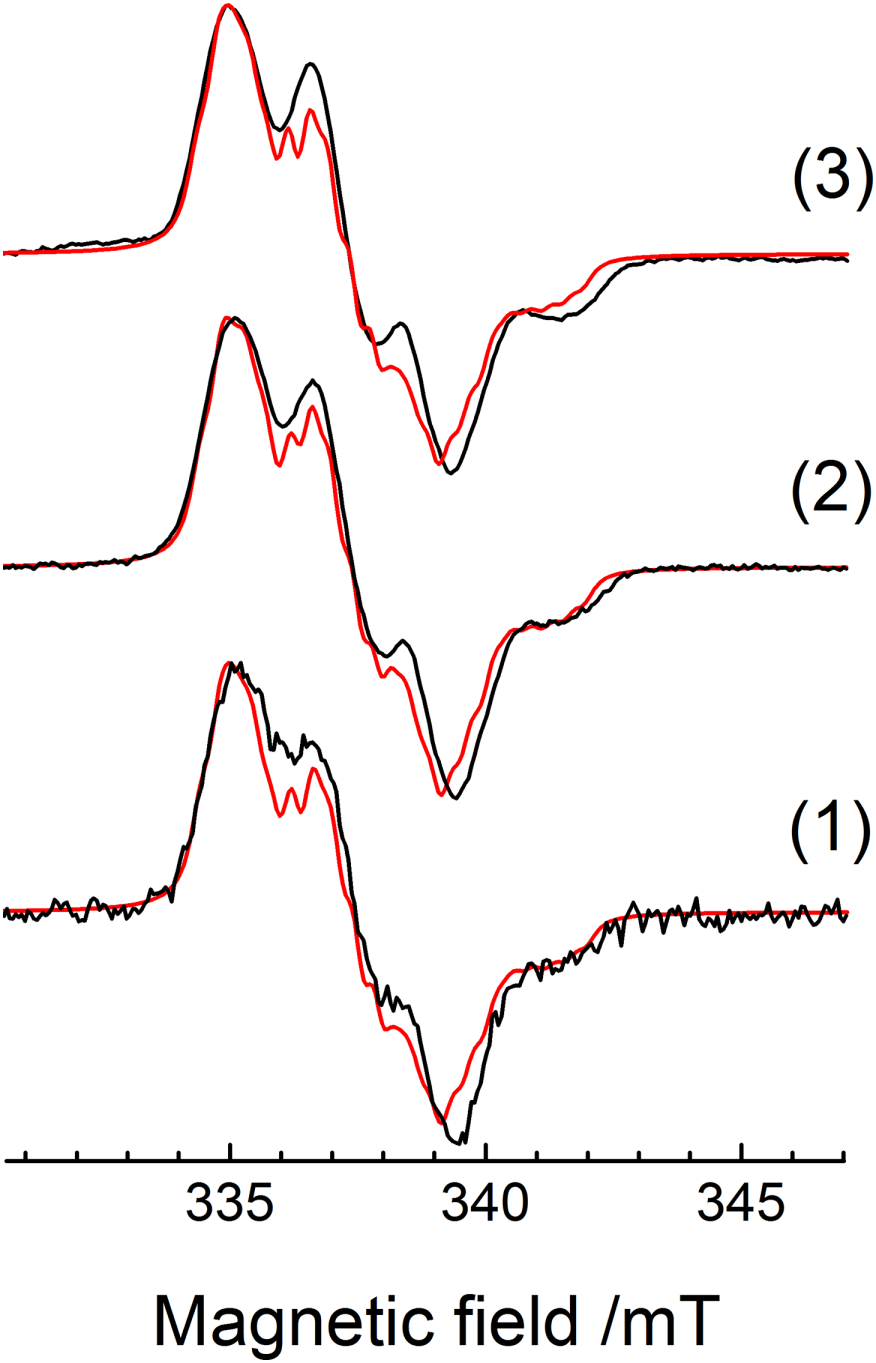
X-band cw-EPR spectrum of samples 1–3 (black curves, from bottom to top) and simulation (red curves) using the parameters obtained from Q-band HYSCORE and EDFS analyses.

The set of EPR parameters obtained from the Q-band HYSCORE and EDFS simulations can be tested by comparison between simulated and experimental X-band EPR spectra. Fig 7 shows this comparison for samples **1**–**3**. The good agreement between the experimental (black curves) and simulated spectra (red curves) shows that the set of parameters obtained by analyzing the Q-band results is capable of reproducing the cw X-band spectra, validating the present analysis.

### Variable temperature X-band ESEEM

**Fig 8:**
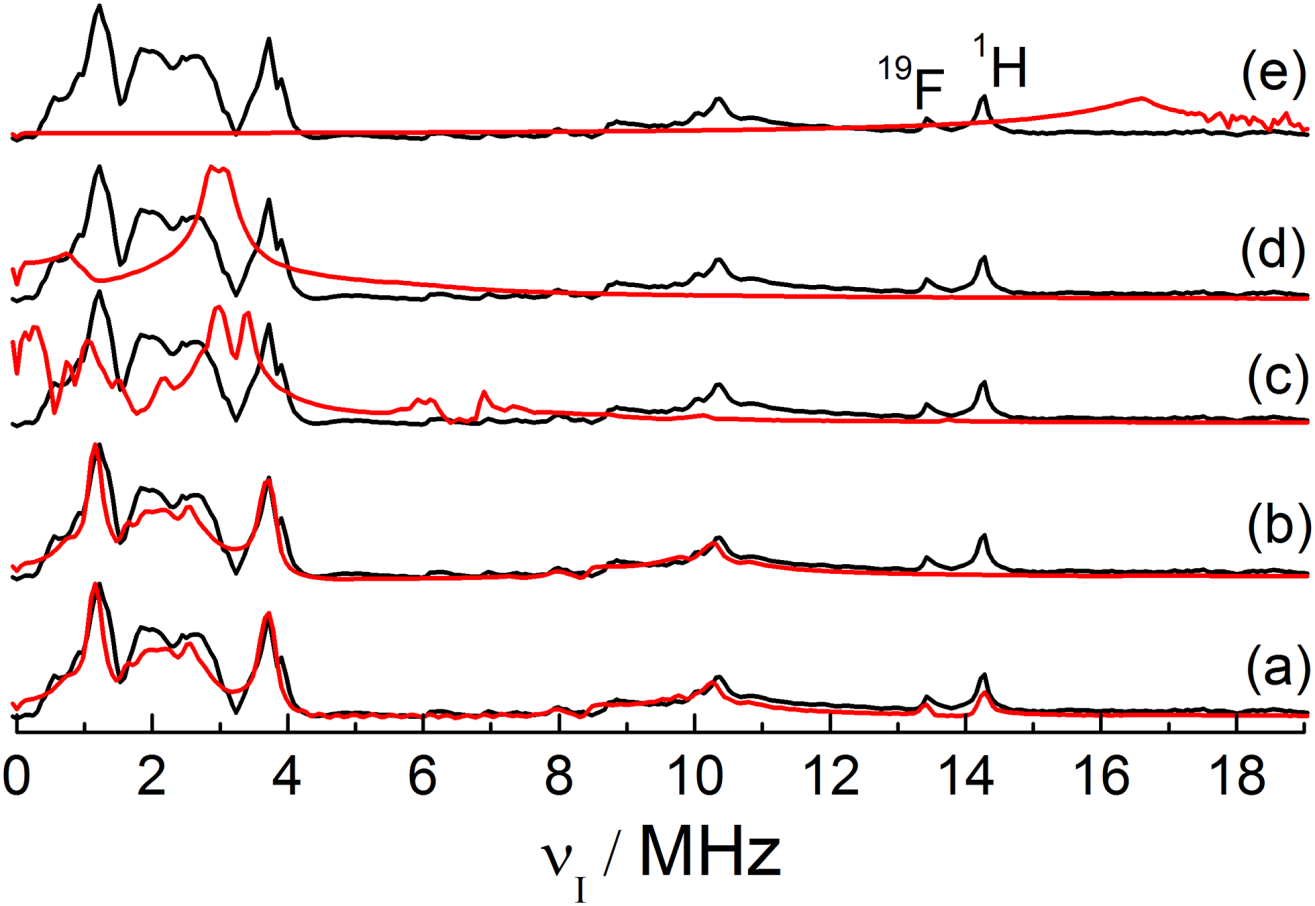
Simulated (red curves) and experimental (black curve) X-band ESEEM spectra for sample 5 obtained with *τ* = 250 ns. The simulations consider different sets of nuclear species interacting with a single unpaired electron. (a) ^10^B, ^11^B, ^14^N, ^31^P, ^19^F, and ^1^H nuclei; (b) ^11^B nucleus only; (c) ^10^B nucleus only; (d) ^14^N nucleus only; and (e) ^31^P nucleus only. The peaks in the ^1^H and ^19^F Zeeman frequencies are labeled in the figure.

Additional insight can be obtained by variable temperature experiments. Since unambiguous simulations for Q-band ESEEM spectra were not obtained, and taking into account that the HYSCORE experiment is very time consuming, the most appropriate experimentis an examination of the effect of temperature variation on the X-band ESEEM spectra. As discussed in the introductory section, in our previous study all the features observed in the ESEEM spectra could be traced to ^11^B hyperfine and ^1^H and ^19^F dipolar interactions (for ^1^H and ^19^F just a sharp signal is observed, located at their Zeeman frequencies at 13.46 and 14.25 MHz, respectively) and ^11^B quadrupolar interactions [8]. We confirmed this fact by means of extensive simulations of different spin systems. The comparison of these simulations (red curves) with the experimental data for sample **5** (black curve) is shown in Fig 8. Part a of this figure shows a simulation considering all the nuclear isotopes present in the FLP system, ^11^B, ^10^B, ^14^N, ^31^P, ^1^H and ^19^F (^15^N was neglected due to the very low abundance). Fig 8b shows a simulation considering only ^11^B hyperfine and quadrupolar couplings. The similarity between the simulations in Fig 8a and 8b (except for the region corresponding to the ^1^H and ^19^F signals) clearly shows that the peak suppression due to the product rule (explained in the introductory section) is perfectly reproduced by the EasySpin [23] simulations. Fig 8c, 8d and 8e show, respectively, simulations considering isolated ^10^B, ^14^N and ^31^P nuclei interacting with the unpaired electron. For all these simulations no match with the experimental data is observed. Therefore, it is reasonable to consider only interactions with the ^11^B isotope in the variable temperature X-band ESEEM analysis. Disregarding the ^1^H and ^19^F species the X-band ESEEM spectra can be simulated using only 9 parameters for ^11^B: the hyperfine tensor components *A*_*xx*_, *A*_*yy*_, and *A*_*zz*_, the quadrupolar coupling parameters *C*_*Q*_ and *η*_*Q*_, the three Euler angles relating the hyperfine and EFG principal axes systems, and a general line broadening parameter.

**Fig 9:**
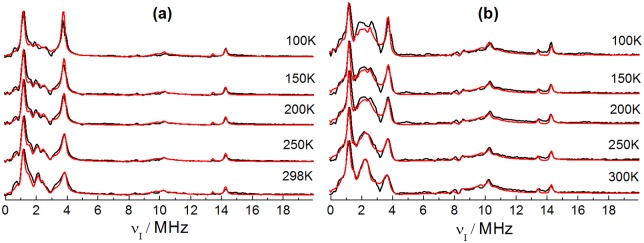
Experimental (black curves) and simulated (red curves) X-band ESEEM spectra obtained with *τ* = 170 ns (a) and *τ* = 250 ns (b) for the sample 5 in the temperature range 100–300 K. The simulations where performed considering isotropic hyperfine coupling tensors for ^1^H and ^19^F nuclei with *A*_*iso*_ = 1.8 MHz and 0.8 MHz respectively, and hyperfine and quadrupolar coupling interactions with ^11^B using the interaction parameters listed in Table 2.

The ESEEM spectra of samples **2–5** were obtained as a function of temperature. The spectra of sample **5** are shown in Fig 9 for two different *τ* values (the corresponding spectra for samples **2–4** are available as S8 to S10 Figs). The features at 0.5–4 MHz, 8 MHz and 10 MHz can be related to hyperfine interactions of the ^11^B nuclei with the unpaired electron. They arise from triple, double and single quantum coherences of the boron spin states in the two electron spin manifolds [8]. The simulations of the spectra recorded at both *τ* values.are based on the same sets of parameters and are in excellent agreement with the experimental data, corroborating the present analysis. Fig 9 also reveals a temperature dependent change in the spectra. Especially a splitting of the signal around 2 MHz is observed from high to low temperatures (more evident in Fig 9b). Besides this change, the relative intensity of the resonance signal at 4 MHz (related to a triple quantum transition) increases, while the peak at 10 MHz is slightly shifted to higher frequencies as the temperature is decreased.

**Fig 10:**
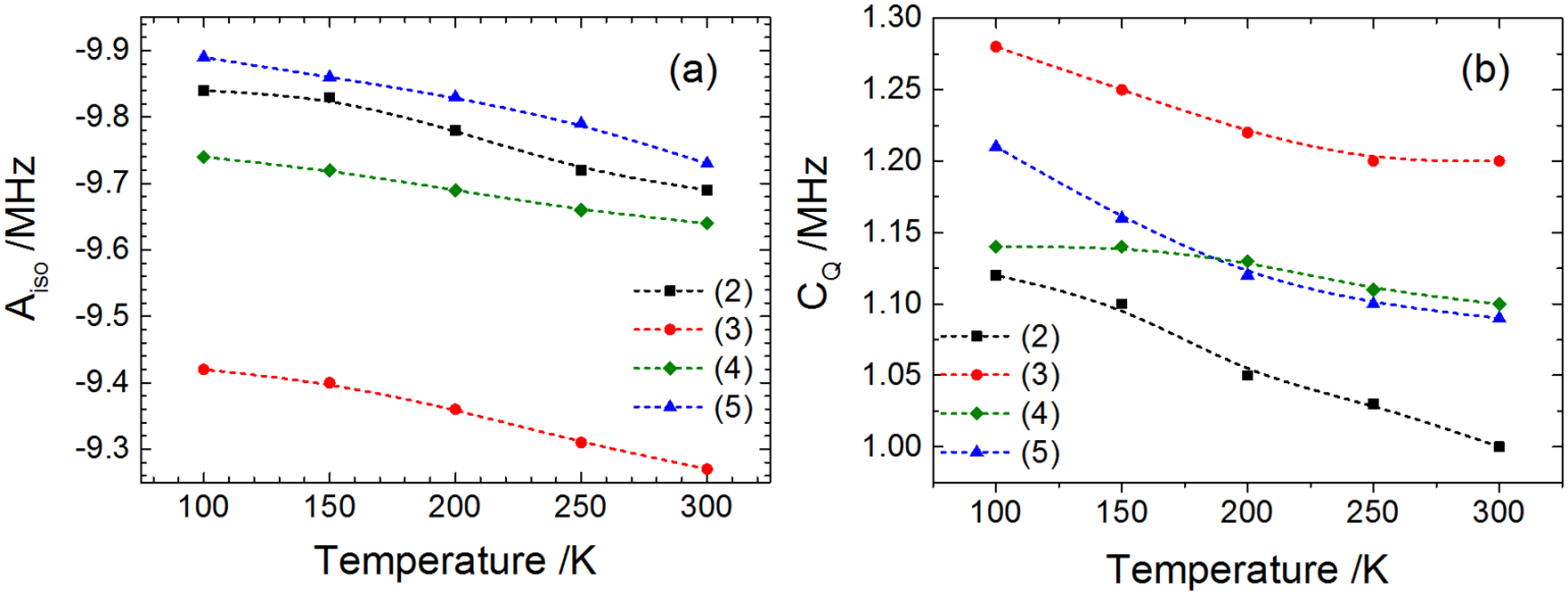
Isotropic ^11^B hyperfine coupling constant (a) and ^11^B quadrupolar coupling constant (b) of samples 2–5 as a function of temperature. The parameters were obtained from simulations of the X-band ESEEM spectra. Dashed lines are drawn as guides for the eyes.

The parameters used for the simulations of samples **2–5** are shown in Table 2. The analysis of the temperature dependence of these parameters reveals, for increasing temperatures, a monotonic decrease of the hyperfine and quadrupolar coupling constants, which is summarized in Fig 10a and 10b. The decrease in the isotropic hyperfine coupling constant reflects a decrease in unpaired electron spin density at the ^11^B nucleus, possibly reflecting an increase in the electron-boron average distance at higher temperatures. This can be explained describing the chemical bonds as anharmonic oscillators. Increased population of excited vibrational levels at higher temperatures leads to longer vibrationally averaged equilibrium distances, which means a decrease in the effective unpaired spin density at the ^11^B nuclear site. The decrease of *C*_*Q*_ with increasing temperature can be explained along similar lines. According to the Bayer theory [24] the increased amplitude of torsional vibrations of the molecule results in increased vibrational averaging of the electric field gradient at the boron position. Overall, the results show that the observed spectral changes with temperature are due to expectable changes in the EPR parameters with temperature, and no specific dynamic phenomena such as structural/conformational rearrangements or phase transitions are observed. As such, the results of this temperature dependent analysis serve to validate the original fitting approach taken in Ref. [8] for the X-band ESEEM spectra.

## Discussion

While the focus of this paper lies on the specific spectroscopic strategy used to solve the multi-parameter fitting problem of the solid state EPR spectra measured for FLP-aminoxyl radicals, a brief discussion of the interpretation of the extracted spin Hamiltonian parameters and their comparison with literature values on related systems is in order. The *g*-tensor values of the present aminoxyl radicals are very similar to those found for the standard nitroxides (such as TEMPO) [10,25], including the orientation of the *g*-tensor relative to the molecular axes. In both the FLP aminoxyl and the standard nitroxide radicals the *z*-axes of the principal coordinate systems of the *g*- and the ^14^N *A*-tensors are found to be colinear [8]. Likewise, the ^14^N quadrupole tensor parameters *C*_Q_ and *η*_Q_ of the present system are very similar to the values reported in the literature for standard nitroxide radicals in frozen solution (*C*_Q_ = 3.18/3.58 MHz and *η*_Q_ = 0.55/0.41) [10]. The ^14^N *C*_Q_ parameter is strongly sensitive to the nature of the NO bonding [26], and the similarity with conventional nitroxide systems indicates that in the FLP aminoxyl radicals this bonding is unaffected if the N-C sigma bonds of the standard nitroxides are replaced by N-B and N-P sigma bonds in the present materials.

Large differences to the standard nitroxides are observed for the ^14^N hyperfine coupling parameters *A*_iso_ and *δ*_A_ [25], however, indicating that the spin densities at the nitrogen atoms are significantly lower in the present radicals. This is understandable, as the large ^31^P and ^11^B hyperfine coupling constants indicate significant electron spin density transfer to these atoms. By normalizing the measured ^11^B and ^31^P isotropic hyperfine coupling parameters with the respective nuclear magnetic moments, the results indicate an ^11^B:^31^P spin density ratio of approximately 1:4. The high spin densities detected at the P atoms may be rationalized by the availability of empty d orbitals through which this spin density may be accommodated.

## Conclusions

In summary, the analyses of the complex nuclear and electronic spin interactions present in nitroxide radicals obtained by N,N addition of nitric oxide to vicinal borane-phosphane Frustrated Lewis Pairs (FLPs) can be made significantly more accurate by adding data from pulsed Q-band EPR spectroscopy in the solid state. The results obtained are highly complementary to those obtained in the previously reported DFT and X-band study [8]. While in the X-band ESEEM and HYSCORE spectra only information about ^11^B hyperfine coupling could be obtained, the corresponding Q-band data contain additional information regarding ^31^P and ^14^N hyperfine couplings. In addition, for the first time experimental information about ^14^N quadrupolar interaction is obtained using EPR techniques for these FLP nitroxide radicals. Specifically, the Q-band echo-detected EPR spectra contain more precise information about the *g* anisotropies than the X-band spectra, which helps to eliminate remaining ambiguities in the previous work for the determination of such parameters.

Within the group of compounds **1–6**, the EPR parameters are very similar to each other, and any differences between them are within the experimental errors associated with the present multi-parameter fitting approach. This finding is not surprising, as the electronic effects of the different backbones upon the electronic densities at the radical center are likely to be very similar in this set of compounds. The effects of more strongly electron donating or withdrawing substituents upon these parameters will be subject of future studies, once such aminoxyl radicals become available.

Variable temperature X-band ESEEM results show excellent agreement between simulated and experimental data, indicating a monotonic decrease of ^11^B hyperfine coupling constants and quadrupolar coupling constants. This effect can be attributed to the influence of thermally activated vibrations upon electron spin densities and electric field gradients at the ^11^B nuclear sites. Altogether, the combination of pulsed and continuous-wave EPR techniques at multiple fields (Q-band and X-band) together with DFT-guided simulations and temperature dependent studies defines a powerful approach for the accurate determination of the relevant spin interaction parameters in these compounds.

## Supporting Information

S1 Fig Contour plots of HYSCORE simulated spectra of an FLP-NO radical. The simulations consider only hyperfine interaction with ^31^P and the *g*-anisotropy. The ^31^P isotropic hyperfine coupling constant *A*_*iso*_ is varied from (a) to (f), assuming respectively the following values: -30 MHz, -45 MHz, -48 MHz, -49 MHz, -50 MHz and -55 MHz. The simulations were performed at Q-band frequencies and a magnetic field of 1.23 T. The unvaried EPR parameters (the g-tensor components and the *A*-anisotropy *δ*_*A*_ and the asymmetry parameter *η*_A_) assume the best-fit values given in Table 1, third column.
(TIF)

S2 Fig Contour plots of HYSCORE simulated spectra of an FLP-NO radical. The simulations consider only hyperfine interaction with ^31^P and the *g*-anisotropy. The *δ*_*A*_-parameter of the ^31^P hyperfine coupling is varied from (a) to (f), assuming respectively the following values: 0, 2 MHz, 4 MHz, 6 MHz, 9 MHz and 10 MHz. The simulations were performed at Q-band frequencies and a magnetic field of 1.23 T. The unvaried EPR parameters (the *g*-tensor components and the *A*_*iso*_ value, and the asymmetry parameter *η*_A_) assume the best fit values shown in Table 1, third column.
(TIF)

S3 Fig Contour plots of HYSCORE simulated spectra of an FLP-NO radical. The simulations consider only the g-anisotropy, the hyperfine interaction with ^14^N and the ^14^N quadrupolar interaction. The *A*_*iso*_-parameter of the ^14^N hyperfine coupling is varied from (a) to (f), assuming respectively the following values: 16 MHz, 17 MHz, 18 MHz, 19 MHz, 20 MHz and 21 MHz. The simulations were performed at Q-band frequencies and a magnetic field of 1.23 T. The unvaried EPR parameters (the *g*-tensor components, the *A*-anisotropy and the ^14^N quadrupolar coupling parameters *C*_Q_ and *η*_Q_) assume the best fit values shown in Table 1, third column.
(TIF)

S4 Fig Contour plots of HYSCORE simulated spectra of an FLP-NO radical. The simulations consider only the *g*-anisotropy, the hyperfine interaction with ^14^N and the ^14^N quadrupolar interaction. The *δ*_*A*_-parameter of the ^14^N hyperfine coupling is varied from (a) to (f), assuming respectively the following values: 36.0 36.8 37.2 37.6 38.4 and 40.0. The simulations were performed at Q-band frequencies and a magnetic field of 1.23 T. The unvaried EPR parameters (the g-tensor components, the *A*_*iso*_ value, and the asymmetry parameter *η*_A_, and the ^14^N quadrupolar interaction parameters *C*_Q_ and *η*_Q_) assume the best fit values shown in Table 1, third column.
(TIF)

S5 Fig Contour plots of HYSCORE simulated spectra of an FLP-NO radical. The simulations consider only the *g*-anisotropy, the hyperfine interaction with ^14^N and the ^14^N quadrupolar interaction. The *C*_*Q*_-parameter is varied from (a) to (f), assuming respectively the following values: 0, 2 MHz, 2.5 MHz, 3 MHz, 3.5 MHz and 4.0 MHz. The simulations were performed at Q-band frequencies and a magnetic field of 1.23 T. The unvaried EPR parameters (the *g*- and *A*-tensor components and the electric field gradient asymmetry parameter *η*_Q_) assume the best fit values shown in Table 1, third column.
(TIF)

S6 Fig Relative root-mean-square (rms) deviations as a function of the ^31^P hyperfine coupling parameters *A*_iso_ (a) and *δ*_A_ (b) for the comparison between HYSCORE experimental and simulated 2D-spectra. The rms calculations were performed only for the spectral region corresponding to the ^31^P signal.
(TIF)

S7 Fig Superimposed contour plots for the comparison between experimental (blue contours) and simulated (green contours) HYSCORE spectra. Part (a) shows simulations performed considering all the relevant isotopes present in the FLP samples (^11^B, ^10^B, ^14^N and ^31^P). Part (b) shows simulation considering only the ^14^N isotope, giving a better visual comparison between experimental and simulated data. The experimental spectrum belongs to sample 5, which has the best signal to noise ratio within the series.
(TIF)

S8 Fig Experimental (black curves) and simulated (red curves) X-band ESEEM spectra obtained with *τ* = 170 ns (a) and *τ* = 250 ns (b) for the sample 2 in the temperature range 100–300 K. The simulations where performed considering isotropic hyperfine coupling tensors for ^1^H and ^19^F nuclei with *A*_*iso*_ = 1.8 MHz and 0.8 MHz respectively, and hyperfine and quadrupolar coupling interactions with ^11^B using the interaction parameters listed in Table 2 (main text).
(TIF)

S9 Fig Experimental (black curves) and simulated (red curves) X-band ESEEM spectra obtained with *τ* = 170 ns (a) and *τ* = 250 ns (b) for the sample 3 in the temperature range 100–300 K. The simulations where performed considering isotropic hyperfine coupling tensors for ^1^H and ^19^F nuclei with *A*_iso_ = 1.8 MHz and 0.8 MHz respectively, and hyperfine and quadrupolar coupling interactions with ^11^B using the interaction parameters listed in Table 2 (main text).
(TIF)

S10 Fig Experimental (black curves) and simulated (red curves) X-band ESEEM spectra obtained with *τ* = 170 ns (a) and *τ* = 250 ns (b) for the sample 4 in the temperature range 100–300 K. The simulations where performed considering isotropic hyperfine coupling tensors for ^1^H and ^19^F nuclei with *A*_iso_ = 1.8 MHz and 0.8 MHz respectively, and hyperfine and quadrupolar coupling interactions with ^11^B using the interaction parameters listed in Table 2 (main text).
(TIF)

S1 Text Description of the approach used to obtain the spin Hamiltonian parameters that result in simulations that best fit the experimental spectra. (DOCX)
