# Supplementary figures and images for: Aminoxyl Radicals of B/P Frustrated Lewis Pairs: Refinement of the Spin-Hamiltonian Parameters by Field- and Temperature-Dependent Pulsed EPR Spectroscopy
Source: PLoS One. 2016 Jun 23;11(6):e0157944. doi: 10.1371/journal.pone.0157944 (PMC4918942; doi:10.1371/journal.pone.0157944)

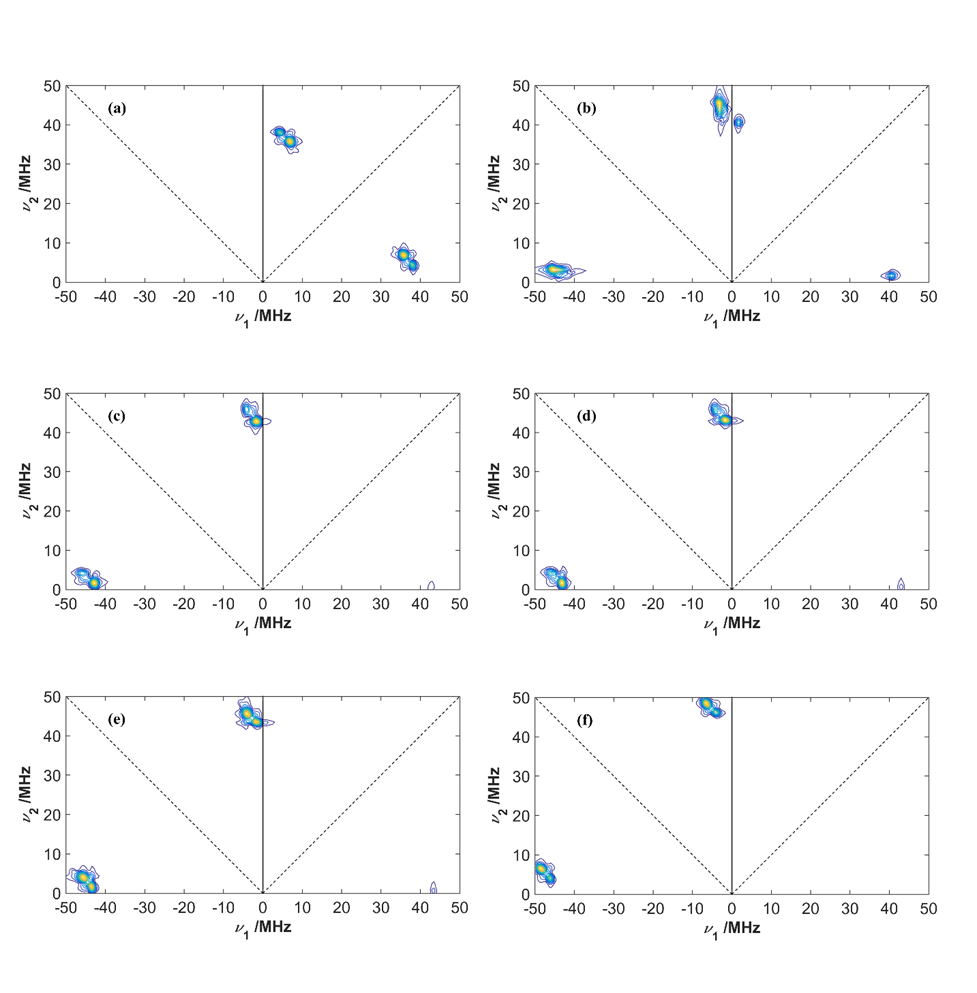

Supplement: S1 Fig — The simulations consider only hyperfine interaction with 31P and the g-anisotropy. The 31P isotropic hyperfine coupling constant Aiso is varied from (a) to (f), assuming respectively the following values: -30 MHz, -45 MHz, -48 MHz, -49 MHz, -50 MHz and -55 MHz. The simulations were performed at Q-band frequencies and a magnetic field of 1.23 T. The unvaried EPR parameters (the g-tensor components and the A-anisotropy δA and the asymmetry parameter ηA) assume the best-fit values given in Table 1, third column. (TIF) [file pone.0157944.s001.tif]

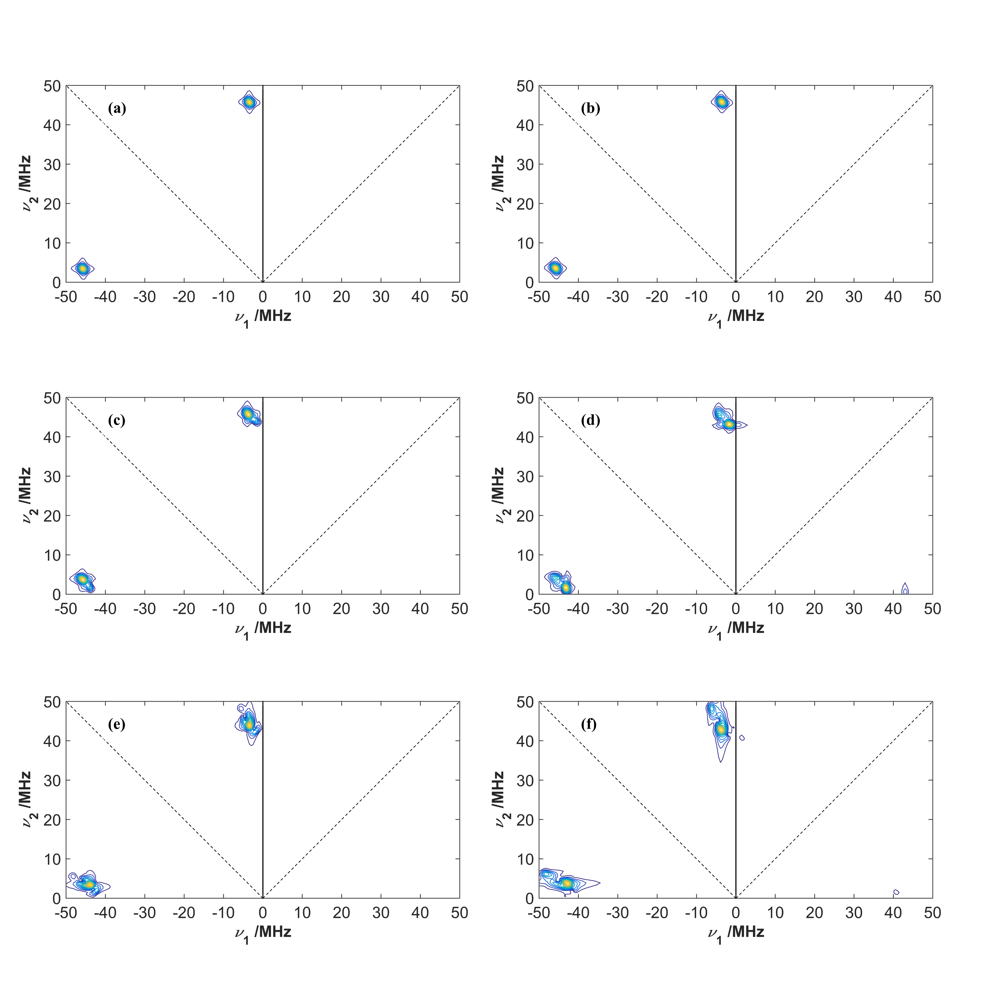

Supplement: S2 Fig — The simulations consider only hyperfine interaction with 31P and the g-anisotropy. The δA-parameter of the 31P hyperfine coupling is varied from (a) to (f), assuming respectively the following values: 0, 2 MHz, 4 MHz, 6 MHz, 9 MHz and 10 MHz. The simulations were performed at Q-band frequencies and a magnetic field of 1.23 T. The unvaried EPR parameters (the g-tensor components and the Aiso value, and the asymmetry parameter ηA) assume the best fit values shown in Table 1, third column. (TIF) [file pone.0157944.s002.tif]

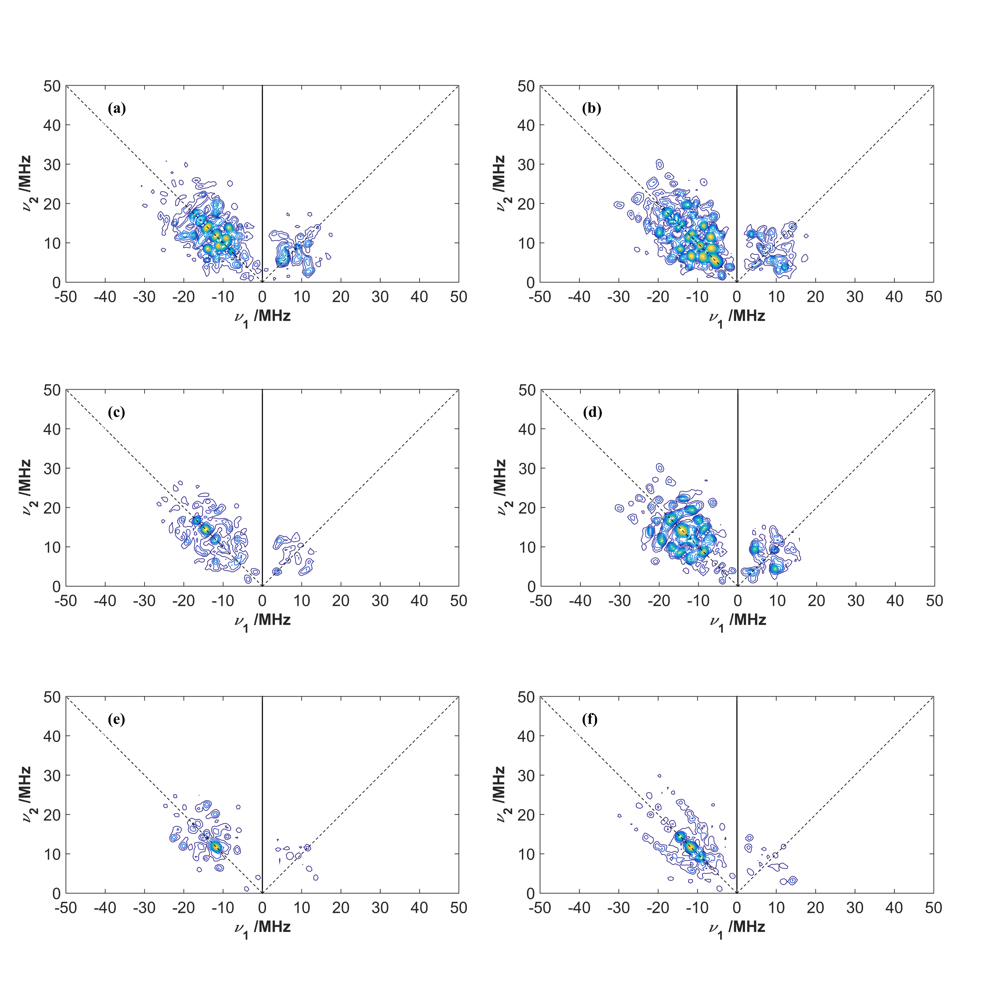

Supplement: S3 Fig — The simulations consider only the g-anisotropy, the hyperfine interaction with 14N and the 14N quadrupolar interaction. The Aiso-parameter of the 14N hyperfine coupling is varied from (a) to (f), assuming respectively the following values: 16 MHz, 17 MHz, 18 MHz, 19 MHz, 20 MHz and 21 MHz. The simulations were performed at Q-band frequencies and a magnetic field of 1.23 T. The unvaried EPR parameters (the g-tensor components, the A-anisotropy and the 14N quadrupolar coupling parameters CQ and ηQ) assume the best fit values shown in Table 1, third column. (TIF) [file pone.0157944.s003.tif]

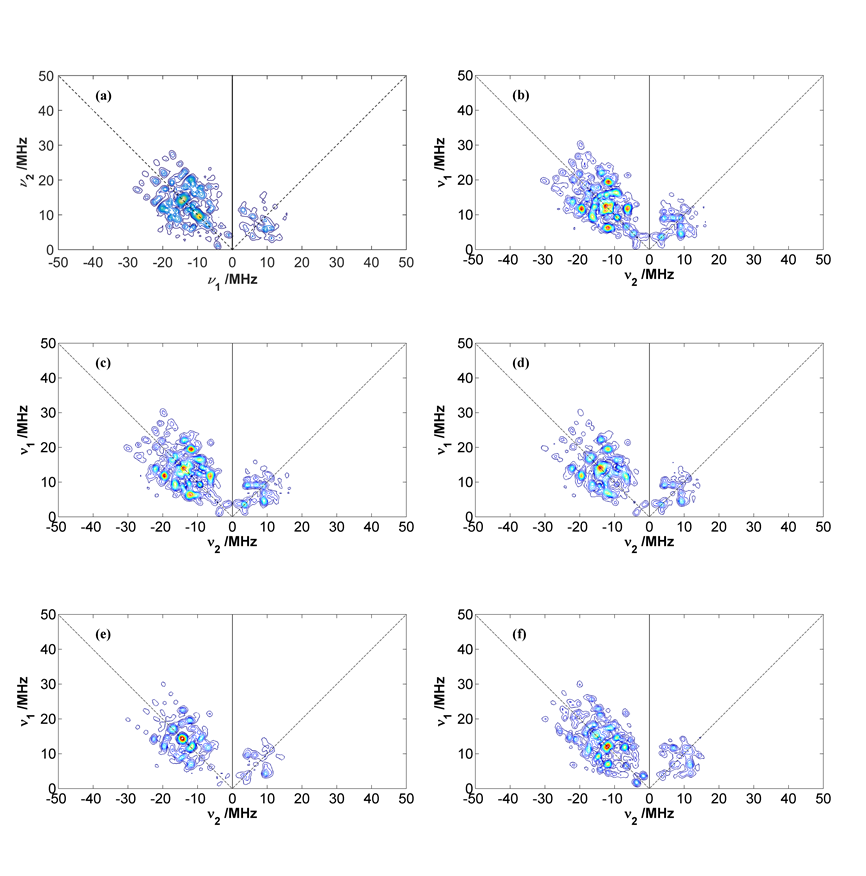

Supplement: S4 Fig — The simulations consider only the g-anisotropy, the hyperfine interaction with 14N and the 14N quadrupolar interaction. The δA-parameter of the 14N hyperfine coupling is varied from (a) to (f), assuming respectively the following values: 36.0 36.8 37.2 37.6 38.4 and 40.0. The simulations were performed at Q-band frequencies and a magnetic field of 1.23 T. The unvaried EPR parameters (the g-tensor components, the Aiso value, and the asymmetry parameter ηA, and the 14N quadrupolar interaction parameters CQ and ηQ) assume the best fit values shown in Table 1, third column. (TIF) [file pone.0157944.s004.tif]

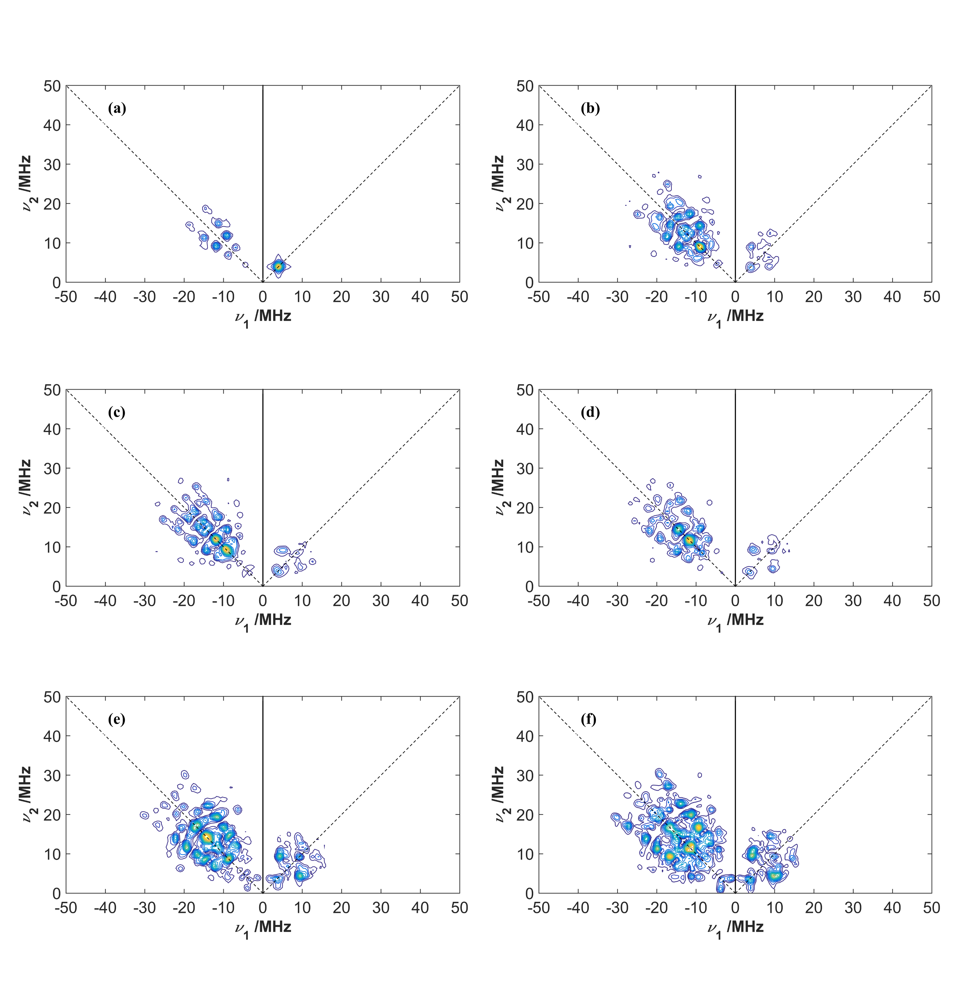

Supplement: S5 Fig — The simulations consider only the g-anisotropy, the hyperfine interaction with 14N and the 14N quadrupolar interaction. The CQ-parameter is varied from (a) to (f), assuming respectively the following values: 0, 2 MHz, 2.5 MHz, 3 MHz, 3.5 MHz and 4.0 MHz. The simulations were performed at Q-band frequencies and a magnetic field of 1.23 T. The unvaried EPR parameters (the g- and A-tensor components and the electric field gradient asymmetry parameter ηQ) assume the best fit values shown in Table 1, third column. (TIF) [file pone.0157944.s005.tif]

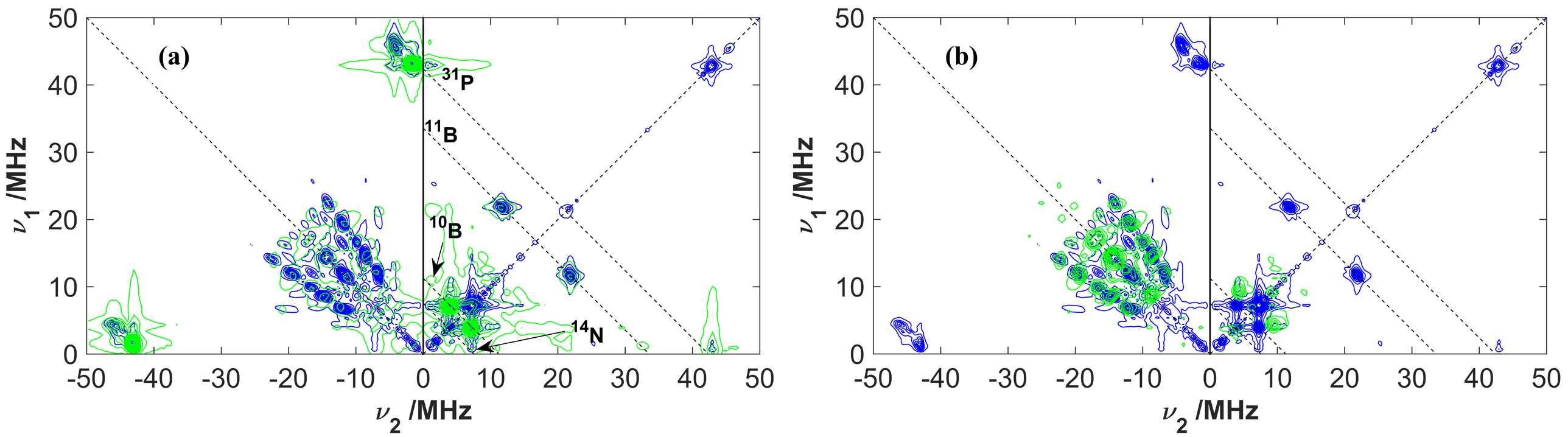

Supplement: S7 Fig — Part (a) shows simulations performed considering all the relevant isotopes present in the FLP samples (11B, 10B, 14N and 31P). Part (b) shows simulation considering only the 14N isotope, giving a better visual comparison between experimental and simulated data. The experimental spectrum belongs to sample 5, which has the best signal to noise ratio within the series. (TIF) [file pone.0157944.s007.tif]
