# Supplementary material for: Aminoxyl Radicals of B/P Frustrated Lewis Pairs: Refinement of the Spin-Hamiltonian Parameters by Field- and Temperature-Dependent Pulsed EPR Spectroscopy
Source: PLoS One. 2016 Jun 23;11(6):e0157944. doi: 10.1371/journal.pone.0157944 (PMC4918942; doi:10.1371/journal.pone.0157944)
Supplement: S1 Text — (DOCX) [file pone.0157944.s011.docx]

**Supplemental Materials**

**Aminoxyl Radicals of B/P Frustrated Lewis Pairs: Refinement of the Spin-Hamiltonian Parameters by Field- and Temperature-Dependent Pulsed EPR Spectroscopy**

**Marcos de Oliveira Jr.,^1^ Robert Knitsch,^2^ Mohammad Sajid,^3^ Annika Stute,^3^ Lisa Elmer,^3^ Gerald Kehr,^3^ Gerhard Erker,^3^ Claudio J. Magon,^1^ Gunnar Jeschke^4^ and Hellmut Eckert^1,2^***

^1^ Instituto de Física de São Carlos, Universidade de São Paulo, P.O. Box 369, 13560-970, São Carlos, São Paulo, Brazil

^2^ Institut für Physikalische Chemie, WWU Münster, Corrensstr. 30, D 48149 Münster, Germany

^3^ Organisch-Chemisches Institut, WWU Münster, Corrensstr. 40, D 48149 Münster, Germany

^4^ Laboratorium für Physikalische Chemie, ETH Zürich, Vladimir-Prelog-Weg 2, 8049 Zürich, Switzerland

* Corresponding author e-mail: [eckerth@uni-muenster.de](mailto:eckerth@uni-muenster.de)

**HYSCORE simulations.** Simulations considering electron spins interacting solely with the ^31^P (Figures S1 and S2) or ^14^N (Figures S3-S5) isotopes were performed in order to identify the signals corresponding to each of the nuclei, to explore the sensitivity of various spectral features toward variations of the parameters *A_iso_* (^31^P), *δ* (^31^P), *A_iso_*(^14^N), *δ* (^14^N), and *C_Q_*(^14^N) parameters. The simulations of Figures S1-S5 clearly show that the spectral regions signifying the effect of ^14^N and ^31^P are very distinct, allowing the unambiguous determination of the EPR parameters by comparison between the simulated and experimental spectra. This was done by performing systematic simulations in which only one EPR parameter was varied, while keeping all the other parameters unchanged. Figures S1 and S2 show these simulations as a function of the ^31^P hyperfine tensor parameters *A_iso_* and *δ_A_*. In order to evaluate the best-fit parameters, the root-mean-square deviation (rmsd) deviation was calculated for each sample as a function of the variable parameters *A_iso_* and *δ_A_*. In doing so, only the spectral region of the experimental HYSCORE spectra showing peaks originating from ^31^P hyperfine coupling were analyzed. The absolute RMS values obtained from these calculations are unrealistic, since the relative peak intensities could not be reproduced by the simulations, which do not consider transversal nuclear relaxation effects. However, an evaluation of the goodness-of-fit could be done by comparing the normalized RMS values, as shown in Figure S6. The plots in this Figure show that within the experimental error the optimum values of *A_iso_* and *δ_A_* are the same for all the compounds (A_iso_ = -49 ± 1 MHz and *δ* = 9 ± 3 MHz). As an example, Figure S7a shows the comparison between the simulated spectrum (green curves) and the experimental one for sample **5** (blue curves). Figures S3-S5 show HYSCORE simulations considering the ^14^N hyperfine parameters *A_iso_*, *δ_A_*, and C_Q_. In this case, the RMS analysis did not yield satisfactory results due to the great complexity of the lineshapes. Therefore, the simulations had to be evaluated by visual inspection using plots similar to the one shown in Figure S7b. As for ^31^P, the ^14^N hyperfine and quadrupolar coupling parameters obtained from the simulations are to be considered identical within experimental error for all compounds (*A_iso_* = 19 ± 1 MHz and *δ_A_* = 56.4 ± 0.2 MHz and *C_Q_* = 3.5 ± 0.5 MHz). The same procedures were adopted for the asymmetry parameters of the hyperfine and quadrupole coupling tensors (not shown). In all cases a strong dependence of the HYSCORE data on the EPR and nuclear interaction parameters is observed, allowing error estimations as to the precision of the simulation parameters.
